# Inhibitory Effects of a Novel Chrysin-Derivative, CPD 6, on Acute and Chronic Skin Inflammation

**DOI:** 10.3390/ijms20112607

**Published:** 2019-05-28

**Authors:** Chan-Hee Yu, Beomseon Suh, Iljin Shin, Eun-Hye Kim, Donghyun Kim, Young-Jun Shin, Sun-Young Chang, Seung-Hoon Baek, Hyoungsu Kim, Ok-Nam Bae

**Affiliations:** 1 College of Pharmacy, Hanyang University, Ansan 15588, Korea; lyu5415@naver.com (C.-H.Y.); christy0615@nate.com (B.S.); rladmsgp615@naver.com (E.-H.K.); ssks7787@naver.com (D.K.); tlsdid24@naver.com (Y.-J.S.); 2 College of Pharmacy, Research Institute of Pharmaceutical Science and Technology, Ajou University, Suwon 16499, Korea; sin214@ajou.ac.kr (I.S.); sychang@ajou.ac.kr (S.-Y.C.); shbaesk@ajou.ac.kr (S.-H.B.)

**Keywords:** skin inflammation, synthetic flavonoid, chrysin, chrysin derivatives, Nrf2/HO-1 signaling

## Abstract

The skin is an important physiological barrier against external stimuli, such as ultraviolet radiation (UV), xenobiotics, and bacteria. Dermal inflammatory reactions are associated with various skin disorders, including chemical-induced irritation and atopic dermatitis. Modulation of skin inflammatory response is a therapeutic strategy for skin diseases. Here, we synthesized chrysin-derivatives and identified the most potent derivative of Compound 6 (CPD 6). We evaluated its anti-inflammatory effects in vitro cells of macrophages and keratinocytes, and in vivo dermatitis mouse models. In murine macrophages stimulated by lipopolysaccharide (LPS), CPD 6 significantly attenuated the release of inflammatory mediators such as nitric oxide (NO) (IC_50_ for NO inhibition: 3.613 μM) and other cytokines. In cultured human keratinocytes, CPD 6 significantly attenuated the release of inflammatory cytokines induced by the combination of IFN-γ and TNF-α, UV irradiation, or chemical irritant stimulation. CPD 6 inhibited NFκB and JAK2/STAT1 signaling pathways, and activated Nrf2/HO-1 signaling. In vivo relevancy of anti-inflammatory effects of CPD 6 was observed in acute and chronic skin inflammation models in mice. CPD 6 showed significant anti-inflammatory properties both in vitro cells and in vivo dermatitis animal models, mediated by the inhibition of the NFκB and JAK2-STAT1 pathways and activation of Nrf2/HO-1 signaling. We propose that the novel chrysin-derivative CPD 6 may be a potential therapeutic agent for skin inflammation.

## 1. Introduction

Skin protects the body from external stimuli, such as xenobiotics, bacteria, and ultraviolet (UV) radiation, and prevents the loss of moisture from the body [1,2]. However, when the skin comes in contact with harmful stimuli, an inflammatory reaction occurs, and the barrier function is gradually lost. Inflammatory reactions, which can be acute or chronic, are characterized by pain, swelling, redness, and heat [3]. An acute inflammatory response is characterized by increased vascular permeability, local vasodilation, and infiltration of immune cells such as macrophages. Chronic inflammation, which is mediated by monocytes and lymphocytes, is a prolonged inflammatory response characterized by tissue destruction [4].

The immune response plays an important role in the host defense against harmful stimulants. However, excessive inflammatory reaction resulting from an impaired immune system can contribute to the development of various skin disorders such as allergic/irritant contact dermatitis, atopic dermatitis, and psoriasis [5,6]. These chronic recurring disorders are characterized by various clinical symptoms of edema and epidermal hyperproliferation [7,8]. The therapeutic options to treat these skin diseases are limited to immunosuppressive agents such as glucocorticosteroids and inhibitors against calcineurin pathway [9]; however, these treatments are reported to be associated with various side effects [10]. Urgent needs exist to develop new anti-inflammatory agents with optimal efficacy and minimal side effects.

The pathophysiological mechanism of skin inflammation is initiated by the activation of cellular pathways, including the nuclear factor κB (NFκB) and mitogen activated protein kinases (MAPK) pathways. The activation of these pathways leads to the release of several pro-inflammatory cytokines of interleukin (IL)-1β, IL-6, and tumor necrosis factor-α (TNF-α) along with the expression of pro-inflammatory enzymes, which involve the release of nitric oxide (NO) and prostaglandin E_2_ (PGE_2_) [5,11,12,13]. Interestingly, nuclear erythroid 2-related factor/heme oxygenase-1 (Nrf2/HO-1), which is an anti-inflammatory signaling pathway, also plays a significant role in modulating the inflammatory response of the skin [14,15]. In addition, considerable attention has been given to the regulation of inducible NO synthase (iNOS) expression via activation of the Janus kinase (JAK2)/signal transducers and activators of transcription 1 (STAT1) signaling pathway [16,17,18,19]. Modulating the STAT1 and corresponding iNOS pathways may be an effective therapeutic approach for treating inflammatory diseases; however, few studies have examined drug candidates that specifically target the regulation of the JAK2-STAT1-iNOS axis. Drug candidates that target these intracellular pathways to modulate the release of inflammatory mediators as therapeutic interventions in skin inflammation should be developed.

The development of novel drugs from natural compounds is safe and beneficial. The use of natural compounds to treat inflammatory diseases has received special attention, as natural compounds have strong anti-oxidant and anti-inflammatory effects. Several flavonoids have been applied as anti-inflammatory agents in acute or chronic inflammatory models [20,21]. These naturally occurring compounds are not necessarily the final drug entity, but rather can serve as sources of novel structures. It is efficient and economically beneficial to study natural products during the developmental stage. Currently, approximately 20% of drug development studies are devoted to developing natural compounds into efficacious modulators [22]. The enhancement of bioactive natural compounds by derivatization is a promising strategy in drug development, but reports on the anti-inflammatory activity of synthetic flavonoid derivatives to treat skin inflammation are still limited.

Recently, Choi et al. reported that chrysin, a naturally occurring flavone, attenuates skin inflammation [23], suggesting the therapeutic potential of chrysin against skin disorders. In this study, we have synthesized several chrysin-derivatives and investigated the anti-inflammatory potential of these compounds using in vitro macrophages and keratinocytes and in vivo dermatitis models.

## 2. Results

### 2.1. Flavonoid and Its Derivatives Inhibit the Release of NO in LPS-Activated Murine Macrophages

**Figure 1:**
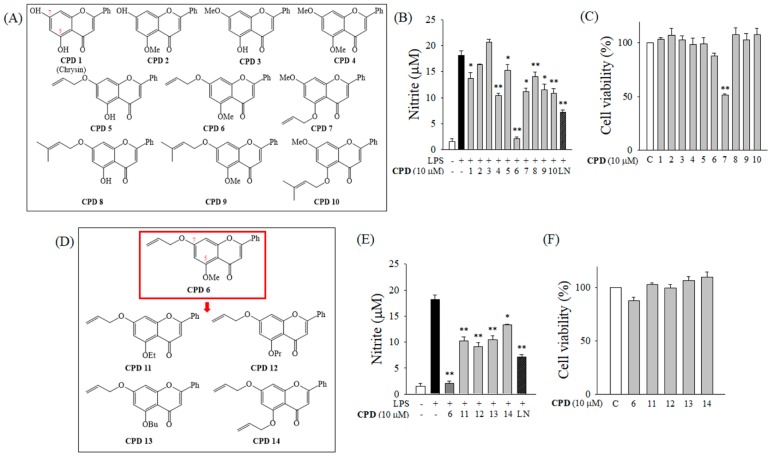
Inhibition of the release of nitric oxide (NO) in lipopolysaccharide (LPS)-activated macrophages by chrysin and its derivatives. (**A**) Chemical structures of chrysin (CPD 1) and its derivatives (CPD 2 to CPD 10) are shown. (**B**) Effect of chrysin and its derivatives on the release of NO was determined by Griess reaction after LPS challenge (200 ng/mL) for 24 h in murine macrophage RAW 264.7 cells. (**C**) Effect of chrysin and its derivatives (CPD 1 to 10) on cell viability was analyzed by an MTT assay after 24 h incubation in RAW 264.7 cells. (**D**) Four additional compounds (CPD 11 to 14), which are derivatives of CPD 6 (Red Box) at atom C (5). (**E**,**F**) Effect of CPD 6 and CPD 11 to 14 on NO accumulation (**E**) or cell viability (**F**) was examined. LN, L-NAME. Data are expressed as mean ± SEM, * *p* < 0.05, ** *p* < 0.01 vs. LPS-treated group. *N* = 3.

**Figure 2:**
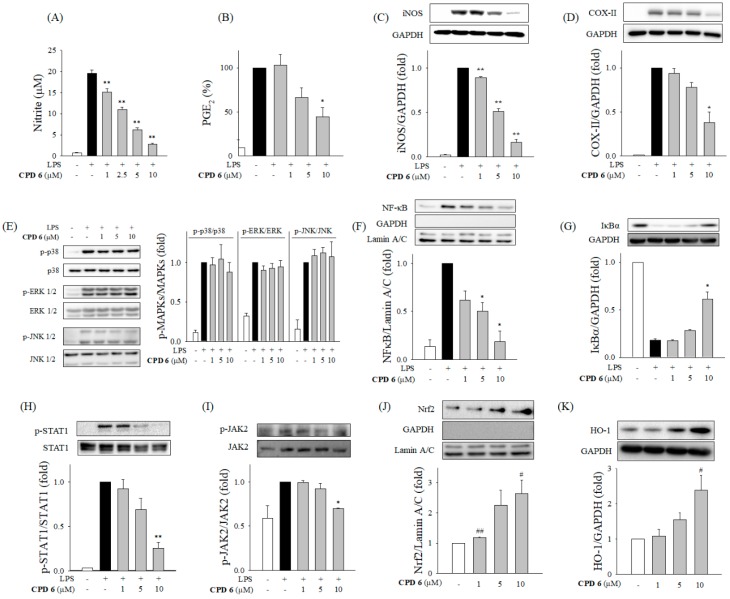
Anti-inflammatory effect of CPD 6 in LPS-activated macrophages and the responsible signaling pathways. (**A** and **B**) Effects of CPD 6 on the release of NO (**A**) and PGE_2_ (**B**) were examined after 24 h LPS challenge in RAW 264.7 cells using the Griess reagent and ELISA, respectively. (**C**,**D**) The protein levels of iNOS (**C**) and COX-II (**D**) were measured by western blot. (**E**) Macrophages were stimulated by LPS for 1 h to determine phosphorylation of the MAPKs (p38, ERK, and JNK) in the presence or absence of CPD 6. (**F**,**G**) Effect of CPD 6 on NFκB nuclear translocation (**F**) or IκBα degradation (**G**) was measured after LPS treatment for 1 h 30 min, respectively. (**H**,**I**) Macrophages were challenged with LPS in the presence or absence of CPD 6 for 6 h to measure phosphorylated level of STAT1 (**H**) and for 2 h to measure phosphorylated JAK2 (**I**). (**J**,**K**) Nrf2 nuclear translocation (**J**) and HO-1 expression (**K**) were measured after CPD 6 treatment without LPS stimulation for 6 h and 24 h, respectively. GAPDH and Lamin A/C were used as internal controls for the cytoplasmic and nuclear fractions, respectively. Data are expressed as mean ± SEM, * *p* < 0.05, ** *p* < 0.01 vs. LPS-treated group. # *p* < 0.05, ## *p* < 0.01 vs. control group. *N* = 3 or 4.

In order to identify a potent anti-inflammatory synthetic flavonoid, we initially evaluated the inhibitory effects of chrysin (CPD 1) and its derivatives (CPD 2 to 10; Figure 1A) on the release of nitric oxide (NO) in murine macrophage RAW 264.7 cells stimulated by lipopolysaccharide (LPS). Among CPD 1 to 10, CPD 6 had the most potent inhibitory effect against LPS-induced NO release without cytotoxicity (Figure 1B,C). Since CPD 6 has a methoxy group at position C (5), we modified the C(5) atom and synthesized four additional derivatives (CPD 11 to 14; Figure 1D). The anti-inflammatory effects of CPD 6 and CPD 11 to 14 were determined based on their inhibitory effects against NO release in LPS-stimulated macrophages, and their representative cytotoxicities were determined by cell viability (Figure 1E,F). Among CPD 6 and CPD11 to 14, CPD 6 remained the most potent, with an IC_50_ value of 3.613 μM (Figure 2A). Based on the NO inhibitory activity of the synthetic flavonoids, CPD 6 was chosen for further examination of its anti-inflammatory activity and the mechanisms.

### 2.2. Inhibitory Effect of CPD 6 on the Release of Inflammatory Mediators from LPS-Stimulated Macrophages

With the selected CPD 6, we examined the anti-inflammatory activity and mechanism in LPS-activated macrophages. LPS stimulates the release of NO and prostaglandin E_2_ (PGE_2_) through upregulated expression of iNOS and cyclooxygenase-II (COX-II), respectively [24]. Release of these mediators is important in the amplification of the inflammatory reaction [19,20]. CPD 6 significantly inhibited the release of NO and PGE_2_ in activated macrophages in a dose-dependent manner (Figure 2A,B). The induction of iNOS and COX-II, as determined by the protein levels, were significantly down-regulated by CPD 6 (Figure 2C,D).

To uncover the responsible mechanisms, we examined the effect of CPD 6 on pro-inflammatory signaling pathways including MAPKs, NFκB, and JAK2/STAT1 [18,25,26]. Each MAPK (p38, ERK1/2, and JNK1/2) was phosphorylated by LPS stimulation, but CPD 6 did not affect the activation of the MAPKs (Figure 2E). The activation of NFκB involves the phosphorylation of NFκB and degradation of IκBα, leading to NFκB nuclear translocation. As shown in Figure 2F,G, LPS stimulation induced significant IκBα degradation and subsequent translocation of NFκB in macrophages, and CPD 6 significantly reduced degradation of IκBα and suppressed NFκB nuclear translocation in LPS-activated macrophages. In orchestrating these pro-inflammatory signaling pathways, JAK2/STAT1 pathway has been reported to be specifically involved in modulating iNOS expression [27,28]. CPD 6 significantly decreased the LPS-induced phosphorylation of STAT1 and JAK2 (Figure 2H,I) in a concentration-dependent manner. Next, the effect of CPD 6 on the stress-related signaling of Nrf2/HO-1 was measured, since this signaling pathway plays a protective role in inflammatory diseases [15]. CPD 6 treatment significantly increased Nrf2 nuclear translocation, consistent with increased HO-1 expression (Figure 2J,K). Taken together, CPD 6 exerted its anti-inflammatory activity by inhibiting the NFκB and JAK2/STAT1 pathways and stimulating Nrf2/HO-1 signaling in macrophages.

### 2.3. CPD 6 Downregulates Inflammatory Mediators in Keratinocytes Stimulated by TNF-α/IFN-γ, UV, or Chemical Irritant

**Figure 3:**
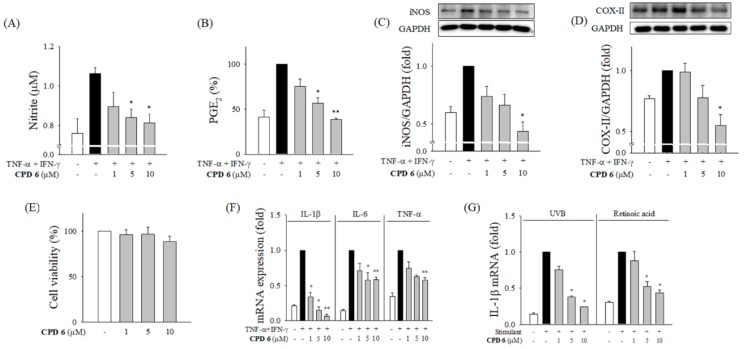
Attenuation of inflammatory signals in CPD 6 in keratinocytes challenged by TNF-α and IFN-γ, UV irradiation, or a chemical irritant. (**A** and **B**) Effect of CPD 6 on the release of NO (**A**) and PGE_2_ (**B**) was measured after 24 h challenge of TNF-α+IFN-γ combination (10 ng/mL each) in human keratinocytes, HaCaT cells. The released amount of NO or PGE_2_ was determined by Griess reaction or ELISA, respectively. (**C**,**D**) The protein levels of iNOS (**C**) or COX-II (**D**) were measured in HaCaT cells activated by TNF-α/IFN-γ. (**E**) Effects of CPD 6 on the cell viability of HaCaT cells were examined by MTT assay. (**F**) The levels of mRNA for IL-1β, IL-6, and TNF-α were measured in human keratinocytes stimulated by TNF-α/IFN-γ using quantitative real time PCR. (**G**) The mRNA levels of IL-1β in keratinocytes were examined at 6 h after UVB irradiation (25 mJ/cm^2^) or exposure to retinoic acid (50 µM). (**G**) Data are expressed as mean ± SEM, * *p* < 0.05, ** *p* < 0.01 vs. TNF-α/IFN-γ or UVB or retinoic acid-treated group. *N* = 3.

Keratinocytes are the first line of defense in skin, but are continuously challenged by various stimuli including bacteria, UV, and chemicals. Keratinocytes play a critical role in initiating the inflammatory response in the skin by releasing pro-inflammatory mediators contributing to skin diseases [12,20]. To examine the therapeutic potential of CPD 6 against skin disorders, we investigated the protective role of CPD 6 in inflammatory responses in human keratinocytes, HaCaT cells. Consistent with the effects in macrophages, CPD 6 significantly reduced the release of NO and PGE_2_ by suppressing iNOS and COX-II expression in keratinocytes stimulated with TNF-α/interferon-gamma (IFN-γ; Figure 3A–D). CPD 6 did not alter the cell viability of HaCaT cells at the concentrations used in the study (Figure 3E), supporting the conclusion that this compound does not induce non-specific cytotoxicity. Notably, the mRNA expression of inflammatory cytokines including IL-1β, IL-6, and TNF-α was significantly decreased by CPD 6 in keratinocytes (Figure 3F). 

One of the most common skin irritants is UV. It is known that UVB at 280–315 nm stimulates the epidermis directly to enhance inflammatory reactions [29]. To investigate whether CPD 6 has protective effects against UVB-induced skin inflammation, the expression levels of pro-inflammatory cytokines were examined in HaCaT cells irradiated with UVB 25 mJ/cm^2^. Additionally, anti-inflammatory potential of CPD 6 in keratinocytes stimulated with retinoic acid, a well-established skin irritant which has been shown to increase IL-1β expression [1], was examined. The mRNA expression levels of IL-1β were significantly decreased by CPD 6 in HaCaT cells challenged with UVB and retinoic acid (Figure 3G).

### 2.4. Suppression of Acute Skin Inflammation Induced by Phorbol 12-Myristate 13-Acetate (TPA) by CPD 6 in Mice

**Figure 4:**
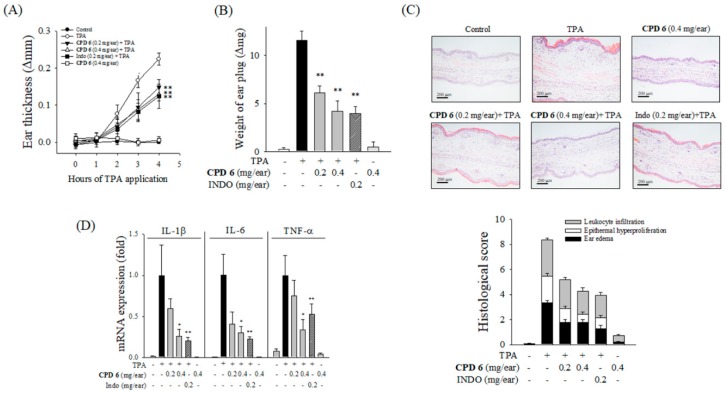
Attenuation of TPA-induced acute skin inflammation by CPD 6 in mice. CPD 6 (0.2 mg or 0.4 mg) or indomethacin (INDO, 0.2 mg) was topically administered to the right ear at 30 min prior to TPA application. (**A**,**B**) The induction of ear edema was assessed by measuring ear thickness (**A**) and ear disc weight (**B**). (**C**) The right ear tissues were examined with H&E staining. Representative histological images from each group were shown (upper panel) and histological scores were plotted (lower panel). Black, white, and gray bars represent ear edema, epithermal hyperproliferation, and leukocyte infiltration, respectively. (**D**) The mRNA levels of IL-1β, IL-6, and TNF-α in the right ear tissue were determined 4 h after TPA application. Data are expressed as mean ± SEM, * *p* < 0.05, ** *p* < 0.01 vs. TPA-treated group. *N* = 5.

To elucidate the in vivo relevance of the protective activity of CPD 6, the mouse model of TPA-induced acute skin inflammation was adopted. Topical application of TPA to the ears of mice significantly increased severe epidermal hyperplasia and edema, and the thickness of ear lesions was increased in a time-dependent manner after TPA stimulation. A single topical administration of CPD 6 (0.2 or 0.4 mg/ear) significantly attenuated TPA-stimulated increase in ear thickness. In particular, 0.4 mg/ear of CPD 6 showed similar efficacy as indomethacin (INDO; 0.2 mg/ear; Figure 4A), a well-established anti-inflammatory drug. The protective effect of CPD 6 was also found in reduced weight of an ear plug at 4 h following TPA application compared to that observed in the TPA-treated group (Figure 4B). In ear discs stained with hematoxylin and eosin (H&E), histopathological examination confirmed the protective role of CPD 6 against acute skin inflammation, as observed in ear edema, epidermal hyperproliferation, and inflammatory cell infiltration into the dermis (Figure 4C and Table 1). In addition, CPD 6 significantly reversed the increased expression levels of pro-inflammatory cytokines of IL-1β, IL-6, and TNF-α (Figure 4D), suggesting that CPD 6 is a potential therapeutic candidate for treating acute inflammation by topical application.

### 2.5. Protective Effect of CPD 6 on Chronic Skin Inflammation Induced by Oxazolone in Mice

**Figure 5:**
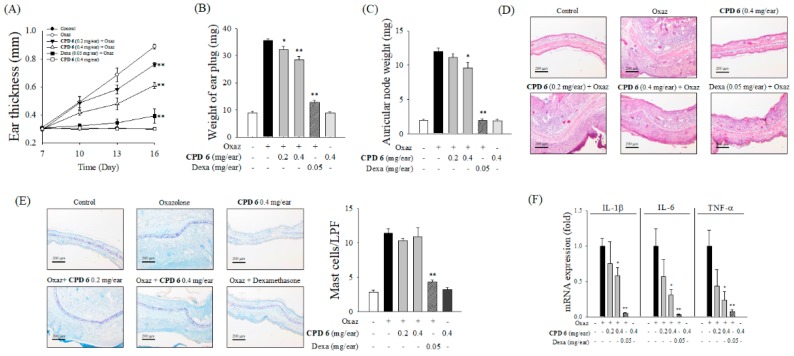
Anti-inflammatory effect of CPD 6 in oxazolone (Oxaz)-induced chronic dermatitis mouse model. On day 0, sensitization was initiated by topical oxazolone treatment on dorsal skin of BALB/c mice, and then inflammatory reaction was induced by challenge with topical oxazolone application on the right ear on days 7, 10, 13, and 16. Thirty minute before and 3 h after each elicitation, CPD 6 (0.2 or 0.4 mg) or dexamethasone (Dexa, 0.05 mg) was applied to the right ear. (**A** to **C**) The changes in ear thickness (**A**), the weight of ear plug (**B**), or auricular lymph node (**C**) were examined at 6 h after the final oxazolone challenge. (**D**) Representative images of H&E staining of right ear tissues from each group are shown (upper panel) with histological scores (lower panel). Scores of ear edema, epithermal hyperproliferation, and leukocyte infiltration were expressed as black, white, and gray bars, respectively. (**E**) Representative histological images of right ear tissue in TB-staining were shown (left panel) and the mast cell counts were plotted (right panel). (**F**) The mRNA levels of pro-inflammatory cytokines in right ear tissues were determined 6 h after the final oxazolone application. Data are expressed as mean ± SEM, * *p* <0.05, ** *p* < 0.01 vs. oxazolone-treated group. *N* = 5.

Next, we investigated the protective effect of CPD 6 on chronic skin inflammation using oxazolone-induced chronic dermatitis model. Repeated topical application of oxazolone led to a sensitization reaction, and progressive symptoms of dermatitis including increased ear thickness, erythema, and dryness appeared [20]. Application of CPD 6 (0.2 or 0.4 mg/ear) showed a significant protection against oxazolone-induced increase in ear thickness (Figure 5A). Oxazolone significantly increased the weight of the ear and lymph node harvested at day 16, but CPD 6 significantly reversed these increase in a dose-dependent manner (Figure 5B,C). Topically applied CPD 6 significantly inhibited the inflammatory reactions induced by oxazolone, including ear thickness, epidermal hyperproliferation, and infiltration of leucocytes in the ear tissues (Figure 5D and Table 2), while CPD 6 had no protective effect on the cutaneous infiltration of mast cells after oxazolone stimulation as observed in Toluidine blue (TB)-stained ear tissues (Figure 5E). Cytokines are the key mediators also in chronic cutaneous inflammation [30]. As shown in Figure 5F, oxazolone induced mRNA expression of pro-inflammatory cytokines of IL-1β, IL-6, TNF-α; however, the induction of these cytokines was significantly attenuated by the topical application of CPD 6 (Figure 5F).

## 3. Discussion

The number of studies on the development of therapeutic agents from natural compounds has recently increased. Natural compounds can be used as drug themselves, but they can also be synthetically derivatized for higher efficacy and lower toxicity [20,22]. Chrysin (5,7-dihydroxyflavone), which is a naturally occurring flavone found in many plants, honey, and propolis, has been extensively studied for its biological activity. The anti-oxidant and chemo-preventive potential of chrysin has been reported suggesting this molecule as a promising drug candidate [31], and Choi et al. have reported that chrysin attenuates atopic dermatitis in mouse models [23], providing an important clue of the therapeutic potential of chrysin against skin inflammation. Based on the bioactivity of chrysin, there have been recent efforts to enhance the pharmacological efficacy and safety via derivatization of this naturally occurring compound [32]. Interestingly, Song et al. recently published that derivatization of chrysin by gamma irradiation resulted in formation of a chrysin derivative, which showed a protective effect against skin inflammation [33], which has a different chemical structure with the synthesized derivatives in our study. The prevalence of skin diseases including atopic dermatitis has been increased worldwide, and the demand for therapeutic options to treat a skin disorder is now increasing [8,34]. As keratinocytes play a key role in skin barrier both in physiological and pathological conditions maintaining homeostasis following challenges with UV, chemical irritants, and inflammatory cytokines [1,3,6], investigation of chrysin and its derivatives in keratinocytes would open a new opportunity for their application in skin diseases. After an initial potency screening of several chrysin-derivatives in macrophages (Figure 1), here we have investigated the protective effects of a synthetic chrysin-derivative CPD 6 against inflammation in vitro macrophages (Figure 2) and keratinocytes (Figure 3) and in vivo dermatitis models (Figure 4 and Figure 5). Further study warrants the details of the structure-activity relationship in the potential of chrysin and chrysin derivatives in different structure against skin inflammation. Comparison of protective potency of chrysin and derivatives against different stimuli such as UV and chemical irritants would be also interesting in terms that keratinocytes are continuously or simultaneously exposed to these stresses [1,2]. 

In our study, CPD 6 significantly inhibited NO production and PGE_2_ synthesis in murine macrophages, demonstrating an anti-inflammatory effect. While chrysin (CPD 1) shows inhibitory effect of 32.9 ± 14.9% (mean ± SD) against LPS-induced NO production, CPD 6 inhibited NO production 96.7 ± 2.1% (mean ± SD) at the same concentration tested (10 µM). In experiments elucidating the underlying mechanisms, CPD 6 attenuated the pro-inflammatory NFκB and JAK2/STAT1 pathways and stimulated the protective Nrf2/HO-1 signaling pathway in macrophages, confirming the anti-inflammatory effects. NFκB translocates to the nucleus and initiates transcriptional activation of the target genes. In our study, we confirmed that CPD 6 reduced IκBα phosphorylation and decreased the translocation of NFκB, thus supporting its anti-inflammatory effect mediated by transcriptional regulation. Along with the NFκB signaling, recent studies have suggested that the JAK2/STAT1 pathway is also important for iNOS expression. JAK2/STAT1 pathway is one of the main inflammatory signaling activated by pro-inflammatory stimuli. When an inflammatory stimulus activates representative cell surface receptor, the receptor-associated JAK2 is phosphorylated. Phosphorylated JAK2 then phosphorylates STAT1, and STAT1 translocates into the nucleus activating the transcription of pro-inflammatory mediators including iNOS [18,28,35,36]. Several phenolic compounds, such as oroxylin A, or coniferyl aldehyde, elicit anti-inflammatory activities in vitro via the inhibition of the JAK2-STAT1 pathway [18,27,37], suggesting that this signaling can be a main target for natural compounds. In this study, CPD 6 also inhibited the JAK2/STAT1 pathway.

It is difficult to point out which pathway is the most primarily responsible target for CPD 6 anti-inflammatory activity. However, multiple modulation of the pro-inflammatory signaling may be beneficial to efficiently inhibit the exacerbation of inflammation. Inflammatory reactions are very complicated phenomena orchestrated by several key pathways including NFκB, MAPK, and STAT1/JAK2 signaling, and these pro-inflammatory signaling pathways were interconnected to regulate each other [14,16,17,38]. While NFκB and JAK2-STAT1 pathways are modulated by CPD 6, the activation of the MAPKs was not affected by CPD 6 (Figure 2E,F), suggesting that anti-inflammatory activity of CPD 6 is not mediated by a non-specific inhibition of inflammatory signaling pathways. Consistent with our observation, previous studies have also reported that endogenous or exogenous molecules modulate these pro-inflammatory effects by inhibiting NFκB, but not MAPK [39,40].

In addition to the pro-inflammatory signaling pathways, the role of anti-inflammatory molecules is also pivotal in inflammatory regulation [14,15]. HO-1 induction through Nrf2 nuclear translocation is a promising approach to treat various inflammatory diseases including atherosclerosis, inflammation induced insulin resistance, and dermatitis [41,42,43]. Here, we showed that CPD 6 regulated Nrf2 translocation and HO-1 expression, demonstrating that Nrf2/HO-1 signaling is one of the main mechanism by which CPD 6 elicits its anti-inflammatory effects. Nrf2 signaling has been recently suggested as one of the main regulator of inflammatory signaling including the regulation of pro-inflammatory signaling of NFκB [44]. Interestingly, activation of Nrf2 and upregulation of HO-1 by CPD 6 was observed at 6 h following CPD 6 treatment without activation of other pro-inflammatory signaling pathways, suggesting that Nrf2/HO-1 signaling may play main role in regulating pro-inflammatory signaling upon inflammatory stimulation. As pointed above, the regulation of these signaling molecules by CPD 6 may be interconnected, but at least as Jung et al. reported that JAK2 and p38 activation is associated with the stimulation of both STAT1 and NFκB signaling [45]. According to Tsoyi et al., HO-1 signaling inhibits the JAK2/STAT1 pathway [19]. The dual modulation mechanism that upregulates Nrf2/HO-1 signaling and inhibits the NFκB pathway has been observed in studies of several compounds, including cinnamaldehyde [46], sulforaphane [47], and macakurzin C-derivative [20]. The crosstalk between these three signaling proteins should be further studied.

Keratinocytes are known to be involved in the induction or amplification of skin inflammation. In our study, inflammatory mediators, such as NO, PGE_2_, and cytokines, were increased as a result of TNF-α and IFN-γ-induced inflammation in keratinocytes. These cytokines play an important role in amplification of skin inflammation. Induction of these inflammatory mediators was significantly suppressed by CPD 6. Besides the amplificatory cytokines such as TNF-α and IFN-γ, skin has been continuously challenged by external stimuli including UV or chemicals. UV radiation is responsible for skin aging, neoplasia, wrinkle formation, and pigmentation. The effects of repeated UV exposure may cause skin disorders, but the even short-term UV irradiation results in burning (erythema) due to skin inflammation and DNA damage [29,48]. Additionally, topically applied chemicals such as retinoic acid can activate the inflammatory response contributing to skin irritation or skin sensitization [1]. Here we demonstrated that CPD 6 significantly attenuated the increase IL-1β mRNA expression induced by UVB or retinoic acid, reflecting that CPD 6 may possess anti-inflammatory activity in keratinocytes regardless of the stimuli.

We have elucidated in vivo protective effects of CPD 6 using two different dermatitis models in mice. The acute skin inflammation model using topical TPA application has been widely used in acute dermatitis research [20]. Typical inflammatory reactions including edema, epidermal hyperplasia, and upregulation of inflammatory cytokines are observed in TPA-induced acute dermatitis model. The oxazolone-induced chronic dermatitis model is a well-established experimental model for studying chronic skin diseases including atopic dermatitis. After sensitization, oxazolone was repeatedly applied to induce chronic inflammation, which is characterized by edema, epidermal hyperplasia, erythema, and leukocyte infiltration, along with the cutaneous recruitment of mast cells [20,40]. Topical application of CPD 6 significantly improved the symptoms of both in acute- and chronic-dermatitis in a dose-dependent manner by reducing the thickness of the ears, the weight of the ear plugs, and improved pathological alteration as observed in the histological score. Additionally, CPD 6 significantly suppressed the expression of cytokines in ears with dermatitis, consistently with our in vitro results.

Interestingly, the inhibitory potential of CPD 6 in the chronic dermatitis model is not as significant as that in the acute model. There was no statistically significant decrease in mast cell counts in the ear plugs, and significant reduction in the weight of the auricular nodes can only be obtained with high-dose CPD 6. In our in vitro cell models and acute dermatitis model, CPD 6 significantly reduced inflammatory signaling and the generation of pro-inflammatory mediators; however, sensitization and mast cell regulation are distinct phenomena, and CPD 6 may not directly regulate these events. The weight of the auricular node is known to correlate with the chemical’s contact sensitivity potential [49]. Steroids have been shown to decrease the auricular node weight increased by sensitization in many previous studies [20]. Vasoactive intestinal peptide (VIP) is the main signaling molecule for mast cell activation [50], and steroids effectively inhibit mast cell activation via the regulation of VIP [51,52]. However, evidence for the anti-sensitizing effect or regulating mast cell activation by the non-steroidal anti-inflammatory drugs (NSAIDs) is limited. In previous studies, indomethacin, one of the representative NSAIDs, did not reduce the auricular node weight in the lymph node analysis [53]. Further studies warrant clarifying the direct effect of CPD 6 on the sensitization process.

In conclusion, our study has shown that CPD 6, a synthesized chrysin-derivative, possess potent anti-inflammatory activities in both murine macrophages and human keratinocytes. This compound inhibited the pro-inflammatory NFκB and JAK2/STAT1 pathways and activated Nrf2/HO-1 signaling. The in vivo experiments demonstrated the anti-inflammatory effects of CPD 6 in both acute- and chronic- dermatitis mouse models. Our results suggest that CPD 6 may be a promising candidate for the treatment of skin inflammation.

## 4. Materials and Methods

### 4.1. Materials

Dimethyl sulfoxide (DMSO), lipopolysaccharide (LPS; *Escherichia coli* O111:B4), MTT (3-(4,5-dimethylthiazol-2-yl)-2,5-diphenyltetrazolium bromide), N-nitro-L-arginine methyl ester hydrochloride (L-NAME), sodium nitrite, Griess reagent, human TNF-α, 2-mercaptoethanol, retinoic acid, phorbol 12-myristate 13-acetate (TPA), 4-ethoxymethylene-2-phenyl-2-oxazolin-5-one (Oxazolone), dexamethasone, indomethacin, and other chemicals were purchased from Sigma-Aldrich (St. Louis, MO, USA). Human IFN-γ was obtained from R&D systems (Minneapolis, MN, USA). Antibodies against iNOS, COX-II, and Nrf2 were purchased from Santa Cruz Biotechnology Inc. (Santa Cruz, CA, USA). Antibody to HO-1 or GAPDH was obtained from Enzo Life Sciences (Plymouth Meeting, PA, USA) or Millipore (Billerica, MA, USA), respectively. Antibodies against NFκB p65, IκBα, phospho-p38 MAPK (Thr180/Tyr182), p38 MAPK, phospho-p44/42 MAPK (Thr202/Tyr204), p44/42 MAPK, phospho-SAPK/JNK (Thr183/Tyr185), SAPK/JNK, phopho-JAK2, JAK2, phospho-STAT1, STAT1, and Lamin A/C were purchased from Cell Signaling Technology (Beverly, MA, USA). Secondary antibodies of horseradish peroxidase (HRP)-conjugated goat anti-mouse IgG and anti-rabbit IgG were obtained from Santa Cruz Biotechnology.

### 4.2. Synthesis of CPD 1 to 14

CPD 1 (chrysin) was purchased from Sigma-Aldrich. Details on the preparation and identification of CPDs 2 to 14 were included in the Supplementary Material.

### 4.3. Cell Culture

Murine macrophage RAW 264.7 cells were acquired from American Type Culture Collection (ATCC, Manassas, VA, USA). Human keratinocyte HaCaT cells were purchased from CLS Cell Line Service (Eppelheim, Germany). Cells were maintained in DMEM (Gibco, Grand Island, NY, USA) supplemented with 10% heat-inactivated FBS (Hyclone, Logan, UT, USA), 100 units/mL of penicillin, and 100 µg/mL of streptomycin (Welgene, Daegu, Korea) at 37 °C under 5% CO_2_ and 95% air in a humidified atmosphere. Experiments were performed with RAW 264.7 (passage number 7~14) or HaCaT (passage number 36~40) cells at confluence of 80% or 90%, respectively.

### 4.4. UV Irradiation

Keratinocytes were seeded and incubated for 24 h and then washed with PBS. Subsequently, CPD 6 dissolved in PBS was added to cells, and the cells were irradiated with UVB at an intensity of 25 mJ/cm^2^ using an UV irradiation chamber in Bio-Sun++ system (Vilber Lourmat, Marne-la-Vallée, France).

### 4.5. Determination of Cell Viability

An MTT assay was used to determine cytotoxicity as described previously [20]. RAW 264.7 (1 × 10^4^ cells/well) or HaCaT (5 × 10^3^ cells /well) were seeded in 96-well plates, and maintained at 37 °C for 24 h or 48 hr, respectively. The cells were treated with test compounds for 24 hr. MTT (final concentration of 0.5 mg/mL) was added, and the cells were incubated for an additional 2 h at 37 °C. After removal of media, the formazan crystals were dissolved using DMSO. The absorbance at 570 nm was measured using a multimode plate reader EnSpire (Perkin Elmer, Waltham, MA).

### 4.6. Determination of the Released Levels of NO and PGE_2_

RAW 264.7 (1 × 10^4^ cells /well) or HaCaT (1 × 10^4^ cells /well) were seeded in 24-well plates and incubated for 24 h at 37 °C. RAW 264.7 or HaCaT cells were challenged with LPS or a combination of IFN-γ and TNF-α, respectively, for 24 h in the presence or absence of CPD 6 (1–10 µM)**.** N-nitro-L-arginine methyl ester hydrochloride (L-NAME; 100 µM), the NO synthase blocker, was used as a positive control. After 24 h, the supernatants were collected and centrifuged briefly. The stable end product of NO, nitrite, was measured as an indicator of NO release. The conditioned medium and Griess reagent were mixed and incubated at room temperature for 15 min. The absorbance at 540 nm was measured using a multimode spectrophotometer EnSpire. Released levels of PGE_2_ in the conditioned medium were determined using an ELISA kit (R&D Systems, Minneapolis, MN, USA company, city, state, country), according to the manufacturer’s instructions.

### 4.7. Mesurement of Protein Levels in Western Blot

In the presence or absence of CPD 6 (1–10 µM), RAW 264.7 or HaCaT cells were stimulated with LPS or a combination of IFN-γ and TNF-α, respectively. Total proteins were extracted from cells using a RIPA buffer with protease inhibitor cocktail (Thermo Scientific, Rockford, IL, USA). Nuclear and cytoplasmic fractions were separated by using NE-PER Nuclear and Cytoplasmic Extraction Reagents (Thermo Scientific, Rockford, IL, USA), when analyzing the translocation of NFκB and Nrf2 to nucleus. The concentrations of proteins were determined by a BCA assay (Thermo Scientific). After being mixed with a Laemmli buffer supplemented 2-mercaptoenthanol, the protein samples were heated at 95 °C for 3 min. The protein samples in equal amounts were loaded and separated in 8% sodium dodecyl sulfate-polyacrylamide gel electrophoresis and transferred onto PVDF membranes (Bio-Rad, Hercules, CA, USA). The membranes were blocked to minimize non-specific antibody binding with 5% (*w*/*v*) skim milk, and then incubated with primary antibodies against inflammatory enzymes (iNOS or COX-II), intracellular signaling molecules (p44/42 MAPK, p38 MAPK, SAPK/JNK, IκBα, HO-1, JAK2, NFκB, Nrf2, or STAT1) or the phosphorylated forms of these signaling molecules (p-p44/42 MAPK, p-p38 MAPK, p-SAPK/JNK, p-JAK2, p-STAT1) at 4 °C overnight. After being washed, the membranes were incubated with the corresponding HRP-conjugated secondary antibodies. The protein signals were visualized using Clarity Western ECL Substrate (Bio-Rad) in the digital imager ChemiDoc XRS System (Bio-Rad). Relative band densities were estimated using ImageJ software. GAPDH was used as a loading control. Lamin A/C was used as an internal marker for nuclear fraction.

### 4.8. Determination of mRNA Levels in Quantitative Real Time PCR (qRT-PCR)

In the presence or absence of CPD 6 (1–10 µM), RAW 264.7 were stimulated with LPS for 6 h and the level of mRNA expression was determined with using qRT-PCR. HaCaT cells were stimulated IFN-γ/TNF-α, retinoic acid or UV. Total RNA was purified using Trizol reagent (Life Technologies, Carlsbad, CA), and the isolated RNA (500 ng) was converted to cDNA by reverse transcription in MyCycler™ Thermal Cycler (Bio-Rad) using ReverTra Ace^®^ qPCR RT kit (Toyobo, Osaka, Japan). qRT-PCR was performed on a CFX96TM Real-Time PCR Detection System (Bio-Rad) using iQTM SYBR Green supermix (Bio-Rad), under the conditions of 95 °C for 10 min, followed by 45 cycles (98 °C for 10 sec, 60 °C for 30 sec, and 72 °C for 1 min), and elongation at 72 °C for 5 min. The primers for qRT-PCR were obtained from GENOTECH (Daejeon, Korea). The levels of mRNA expression were calculated using the comparative CT method (2^−∆∆Ct^) with normalization by the level of GAPDH. The sequences for mouse primers, which were used for RNA from murine macrophages, were as follows: For IL-1β, sense 5′-CAACCAACAAGTGATATTCTCCATG-3′ and anti-sense 5′-GATCCACACTCTCCAGCTGCA-3′; for IL-6, sense 5′- AACGATGATGCACTTGCAGA-3′ and anti-sense 5′-GAGCATTGGAAATTGGGGTA-3′; for TNF-α, sense 5′-ATGAGCACAGAAAGCATGATC-3′ and anti-sense 5′-TACAGGCTTGTCACTCGAATT-3′; and for GAPDH, sense 5′-TGAACGGGAAGCTCACTGG-3′ and anti-sense 5′-TCCACCACCCTGTTGCTGTA-3′. The sequences for human primers for human keratinocytes were as follows: For IL-1β, sense 5′-AGGCCTCTCTCACCTCTCCT-3′ and anti-sense 5′-AGAATGTGGGAGCGAATGAC-3′; for IL-6, sense 5′-AAAGAGGCACTGGCAGAAAA-3′ and anti-sense 5′-TTTCACCAGGCAAGTCTCCT-3′; for TNF-α, sense 5′-AGCCCATGTTGTAGCAAACC-3′ and anti-sense 5′-GGCACCACCAACTGGTTATC-3′; and for GAPDH, sense 5′-ACTTTGGTATCGTGGAAGGAC-3′ and anti-sense 5′-AGTAGAGGCAGGGATGATGTT-3′.

### 4.9. Animals

All animal experiments were performed in accordance with the guidelines under the approval of the Institutional Animal Care and Use Committee (IACUC) of Hanyang University, Republic of Korea. Project identification codes are HYUIACUC-20170039A (date of approval: 1 April 2017) and HYUIACUC-20170067A (date of approval: 14 May 2017) for TPA-induced acute ear edema and oxazolone-induced chronic dermatitis, respectively. Thirty male ICR mice or thirty BALB/c mice (six weeks old) were purchased from KOATECH (Gyeonggi, Republic of Korea). Five mice per each experimental group of total six groups were used in each experiment. The mice were acclimated for at least seven days prior to each experiment. The animals were maintained under standard laboratory conditions of 12 h light: 12 h dark cycle at 25 ± 2 °C under relative humidity of 45 ± 5%.

### 4.10. Mouse Models of Acute Ear Inflammation Induced by TPA

In vivo anti-inflammatory activity of CPD 6 against acute skin inflammation was examined in the mouse model of TPA-induced ear edema [20]. Vehicle (20 µL of acetone), CPD 6 (0.2 or 0.4 mg/ear), or indomethacin (0.2 mg/ear) was carefully applied topically to the inner and outer surfaces of right ear 30 min prior to TPA stimulation (2 µg/ear). The corresponding left ear was used as an internal control. The thickness of each ear was determined using a digital caliper (Blue Bird, Seoul, Korea) at every one hour after TPA application. The mice were sacrificed at four hours after TPA application, and ear discs of the central portion of both ears (6 mm in diameter) were collected using a biopsy punch (Kai Industries, Tokyo, Japan). The weight of each ear disc was recorded. The difference in the thickness or in the disc weight between the right ear (TPA application) and the left ear (internal control) was used to determine the extent of edema. Ear samples were fixed in 4% paraformaldehyde for histological examination. After embedded in paraffin, cross sections (6 µm) of ear samples were stained with H&E. The pathological scoring was conducted by an independent histologist, who was blinded to the group assignment. The extent of ear edema (0–5 scale), epidermal hyperproliferation (0–5 scale), and leukocyte infiltration (0–5 scale) was scored as described previously [20]. To measure the mRNA expression of cytokines in ear samples, the right ear discs were homogenized in Trizol reagent. The overall procedures for qRT-PCR including total RNA extraction, cDNA synthesis, and qRT-PCR were conducted as mentioned above.

### 4.11. Mouse Models of Chronic Dermatitis Induced by Oxazolone

Chronic atopic dermatitis model using oxazolone was established as described previously with minor modifications [40]. On Day 0, BALB/c mice were sensitized by a single application of oxazolone (1.5% in ethanol, 100 µL) on the shaved dorsal skin. On Day 7, the mice were challenged by application of 1% oxazolone in a mixture of acetone and olive oil (4:1) on both sides of the right ear (20 µL). The challenge was repeated every three days on Day 10, 13, or 16. To examine the effect of CPD 6 or dexamethasone, vehicle, CPD 6 (0.2 or 0.4 mg/ear) or dexamethasone (0.05 mg/ear) was administered on both sides of the right ear 30 min before and 3 h after each oxazolone stimulation on Day 7, 10, 13, or 16. The thickness of right ear was determined using a digital caliper (Blue Bird) on each day of the oxazolone challenge. The animals were sacrificed at six hours after the final oxazolone challenge on Day 16, and the right ear discs (6 mm in diameter) were collected using a biopsy punch and weighed. The auricular lymph nodes were collected by ventral dissection and the weight was recorded. The ear biopsies were fixed in 4% paraformaldehyde for histological observation. After being embedded in paraffin, cross sections of ear samples were stained with H&E or TB. The levels of cytokine mRNA in ear tissues was measured by qRT-PCR as described above.

### 4.12. Statistical Analysis

Data in figures were expressed as mean ± standard error of mean (SEM) for all experimental groups. Data in tables were expressed as the means ± standard deviation (SD). The statistically significant differences were calculated by an appropriate statistical tests, such as a student’s *t*-test, Mann–Whitney U test, and one-way ANOVA (analysis of variance) with a post-hoc Tukey’s test. Statistical analysis was conducted using SPSS software (Chicago, IL, USA). For all analyses, a *p* value <0.05 was considered statistically significant.

## Figures and Tables

**Table 1:** Effect of CPD 6 on histological observations in TPA-induced acute dermatitis in mice.

Groups	Histological Score (Mean ± S.D.)		
	Ear Edema	Epidermal Hyper Proliferation	Leucocyte Infiltration
Control	0.03 ± 0.129	0.03 ± 0.129	0.00 ± 0.000
TPA	3.33 ± 0.772	2.13 ± 0.767	2.90 ± 0.507
CPD 6 (0.2 mg/ear) + TPA	1.80 ± 0.841 **	1.10 ± 0.632 **	2.30 ± 0.21
CPD 6 (0.4 mg/ear) + TPA	1.77 ± 0.942 **	0.67 ± 0.645 **	1.83 ± 1.097 **
Indo (0.2 mg/ear) + TPA	1.27 ± 1.033 **	0.87 ± 0.767 **	1.80 ± 0.841 **
CPD 6 (0.4 mg/ear)	0.17 ± 0.244	0.07 ± 0.176	0.47 ± 0.481

Statistical significance was determined using a one-way ANOVA (Analysis of Variance) followed by Tukey’s test. Indo, Indomethacin; TPA, phorbol 12-myristate 13-acetate. * *p* < 0.05, ** *p* < 0.01 vs. TPA-treated group. *N* = 5.

**Table 2:** Effect of CPD 6 on histological inflammatory observations in oxazolone-induced chronic dermatitis.

Groups	Histological Score (mean ± S.D.)		
	Ear Edema	Epidermal Hyper Proliferation	Leucocyte Infiltration
Control	0.00 ± 0.000	0.00 ± 0.000	0.00 ± 0.000
Oxaz	4.00 ± 0.667	4.13 ± 0.447	4.8 ± 0.183
CPD 6 (0.2 mg/ear) + Oxaz	3.07 ± 0.830	3.07 ± 0.683 **	3.60 ± 1.140 *
CPD 6 (0.4 mg/ear) + Oxaz	2.47 ± 0.869 **	2.60 ± 0.279 **	3.20 ± 0.558 **
Dexa (0.2 mg/ear) + Oxaz	0.67 ± 0.471 **	0.93 ± 0.596 **	0.73 ± 0.494 **
CPD 6 (0.4 mg/ear)	0.00 ± 0.000	0.07 ± 0.149	0.00 ± 0.000

Statistical significance was calculated using a one-way ANOVA followed by Tukey’s test. Dexa, dexamethasone; Oxaz, oxazolone. * *p* < 0.05 vs, ** *p* < 0.01 vs. Oxaz-treated group. *N* = 5.

## Supplementary Materials

Supplementary materials can be found at https://www.mdpi.com/1422-0067/20/11/2607/s1.

## Abbreviations

COX-II	Cyclooxygenase II
CPD 6	Compound 6
Dexa	Dexamethasone
DMSO	Dimethyl sulfoxide
ERK	Extracellular signal-regulated kinase
GAPDH	Glyceraldehyde 3-phosphate dehydrogenase
H&E	Hematoxylin and eosin
INF-γ	Interferon-gamma
IL-1β	Interleukin-1β
IL-6	Interleukin-6
INDO	Indomethacin
iNOS	Inducible nitric oxide synthase
JAK	Janus kinase
JNK	c-Jun N-terminal kinase
L-NAME	N-nitro-L-arginine methyl ester hydrochloride
LPS	Lipopolysaccharide
MAPKs	Mitogen activated protein kinase
MTT	3-(4,5-dimethylthiazol-2-yl)-2,5-diphenyltetrazolium bromide
NFκB	Nuclear factor kappa B
NO	Nitric oxide
Nrf2	Nuclear factor-erythroid 2-related factor-2
Oxaz	Oxazolone (4-ethoxymethylene-2-phenyl-2-oxazolin-5-one)
PGE_2_	Prostaglandin E_2_
STAT	Signal Transducers and Activators of Transcription
TB	Toluidine blue
TNF-α	Tumor necrosis factor-alpha
TPA	Phorbol 12-myristate 13-acetate
