# Supplementary material for: Inhibitory Effects of a Novel Chrysin-Derivative, CPD 6, on Acute and Chronic Skin Inflammation
Source: Int J Mol Sci. 2019 May 28;20(11):2607. doi: 10.3390/ijms20112607 (PMC6600461; doi:10.3390/ijms20112607)

Supplementary Material

Inhibitory effects of a novel chrysin-derivative, CPD 6, on acute and chronic skin inflammation

Chan-Hee Yu [1,a], Beomseon Suh [1,a], Iljin Shin [2], Eun-Hye Kim [1], Donghyun Kim [1], Young-Jun Shin [1], Sun-Young Chang [2], Seung-Hoon Baek [2], Hyoungsu Kim [2,*] and Ok-Nam Bae [1,*]

[1] College of Pharmacy Institute of Pharmaceutical Science and Technology, Hanyang University, Ansan, Republic of Korea

[2] College of Pharmacy and Research Institute of Pharmaceutical Science and Technology (RIPST), Ajou University, Suwon, Republic of Korea

[a] C.-H. Yu and B. Suh contributed equally.

* Correspondence: hkimajou@ajou.ac.kr (H. Kim); onbae@hanyang.ac.kr, +82-31-400-5805 (O.-N. Bae)

Synthesis of CPD 1-14

CPD 1 (chrysin) was purchased from Sigma-Aldrich (MO, USA). **CPDs 2-8** and **CPD14** were synthesized by according to the literature procedure [1–7]. **CPD 9** and **CPD 10** were prepared from **CPD 8** and **CPD 3** by methylation and prenylation, respectively (Scheme 1). **CPDs 6** and **11–13** were prepared from **CPD 5** by alkylation (methylation, ethylation, *n*-propylation, and *n*-butylation).

Scheme 1. Preparation of **CPDs 6, 9–13**

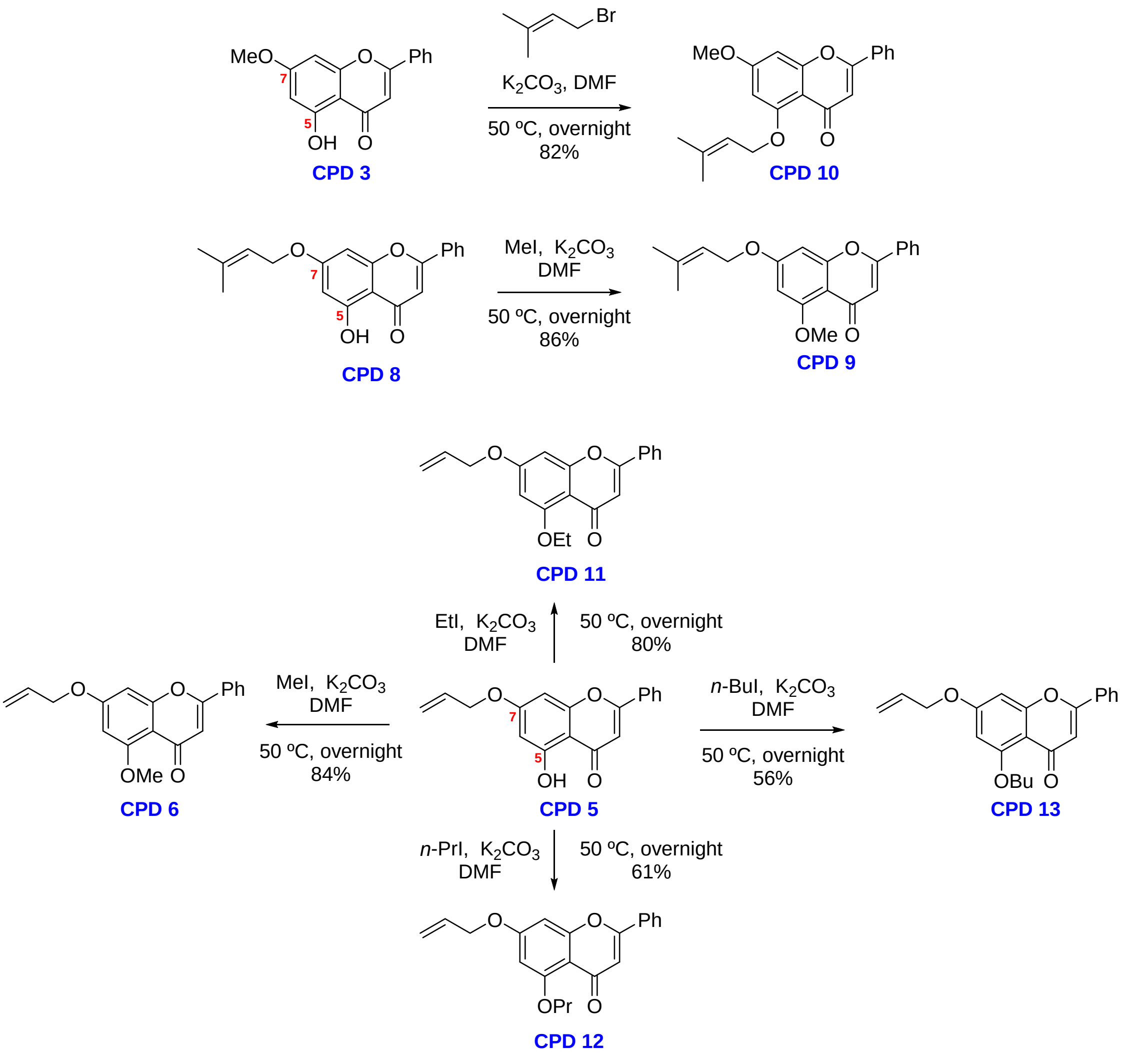

CPD-6: m.p 128.1 °C; ^{1}H NMR (600 MHz, $CDCl_3$) δ 7.89-7.85 (m, 2 H), 7.52-7.48 (m, 3 H), 6.69 (d, *J* = 0.6 Hz, 1 H), 6.58 (d, *J* = 2.4 Hz, 1 H), 6.43 (d, *J* = 2.4 Hz, 1 H), 6.09 (dddd, *J* = 17.4, 10.8, 5.4, 5.4 Hz, 1 H), 5.48 (dddd, *J* = 17.4, 1.8, 1.8, 1.8 Hz, 1 H), 5.37 (dddd, *J* = 10.8, 1.2, 1.2, 1.2 Hz, 1 H), 4.65 (ddd, *J* = 13.2, 7.8, 4.8 Hz, 2 H), 3.97 (s, 3 H); ^{13}C NMR (150 MHz, $CDCl_3$) δ 177.6, 163.0, 161.0, 160.7, 159.8, 132.1, 131.2, 128.9, 126.0, 118.6, 109.4, 109.1, 96.6, 93.7, 69.3, 56.5.

CPD-9: m.p 121.2 °C; ^{1}H NMR (600 MHz, $CDCl_3$) δ 7.90-7.85 (m, 2 H), 7.54-7.46 (m, 3 H), 6.68 (s, 1 H), 6.59 (d, *J* = 2.4 Hz, 1 H), 6.40 (d, *J* = 2.4 Hz, 1 H), 5.53 (dddd, *J* = 7.2, 7.2, 1.2, 1.2 Hz, 1 H), 4.61 (d, *J* = 7.2 Hz, 2 H), 3.95 (s, 3 H), 1.84 (s, 3 H), 1.80 (s, 3 H); ^{13}C NMR (150 MHz, $CDCl_3$) δ 177.6, 163.3, 160.9, 160.6, 159.9, 139.6, 131.6, 131.1, 128.9, 126.0, 118.5, 109.3, 109.1, 96.7, 93.5, 65.3, 56.4, 25.8, 18.3.

CPD-10: m.p 138.3 °C; ^{1}H NMR (600 MHz, $CDCl_3$) δ 7.91-7.85 (m, 2 H), 7.54-7.45 (m, 3 H), 6.65 (s, 1 H), 6.56 (dd, *J* = 2.4, 0.6 Hz, 1 H), 6.38 (d, *J* = 2.4 Hz, 1 H), 5.58 (dddd, *J* = 6.6, 6.6, 1.2, 1.2 Hz, 2 H), 4.69 (d, *J* = 6.6 Hz, 2 H), 3.90 (s, 3 H), 1.79 (s, 3 H), 1.76 (s, 3 H); ^{13}C NMR (150 MHz, $CDCl_3$) δ 177.4, 163.8, 160.5, 160.2, 159.9, 137.4, 131.7, 131.1, 128.9, 125.9, 119.5, 109.7, 109.1, 97.6, 92.9, 66.6, 55.7, 25.8, 18.4.

CPD-11: m.p 146.3 °C; ^{1}H NMR (600 MHz, $CDCl_3$) δ 7.89-7.86 (m, 2 H), 7.52-7.47 (m, 3 H), 6.65 (s, 1 H), 6.56 (d, *J* = 2.4 Hz, 1 H), 6.41 (d, *J* = 2.4 Hz, 1 H), 6.08 (dddd, *J* = 17.4, 10.8, 5.4, 5.4 Hz, 1 H), 5.47 (dddd, *J* = 17.4, 1.8, 1.8, 1.8 Hz, 1 H), 5.37 (dddd, *J* = 10.8, 1.2, 1.2, 1.2 Hz, 1 H), 4.63 (ddd, *J* = 5.4, 1.8, 1.8 Hz, 2 H), 4.16 (ddd, *J* = 7.2, 7.2, 7.2 Hz, 2 H), 1.56 (dd, *J* = 7.2, 7.2 Hz, 3 H); ^{13}C NMR (150 MHz, $CDCl_3$) δ 177.5, 162.9, 160.5, 160.3, 159.8, 132.2, 131.7, 131.1, 128.9, 126.0, 118.5, 109.6, 109.1, 97.5, 93.6, 69.2, 65.0, 14.5.

CPD-12: mp 112.6 °C; ^{1}H NMR (600 MHz, $CDCl_3$) δ 7.89-7.85 (m, 2 H), 7.53-7.46 (m, 3 H), 6.63 (s, 1 H), 6.55 (d, *J* = 2.4 Hz, 1 H), 6.40 (d, *J* = 2.4 Hz, 1 H), 6.08 (dddd, *J* = 17.4, 10.8, 5.4, 5.4 Hz, 1 H), 5.47 (dddd, *J* = 17.4, 1.8, 1.8, 1.8 Hz, 1 H), 5.36 (dddd, *J* = 10.8, 1.2, 1.2, 1.2 Hz, 1 H), 4.63 (ddd, *J* = 5.4, 1.8, 1.8 Hz, 2 H), 4.03 (dd, *J* = 6.6, 6.6 Hz, 2 H), 1.96 (ddddd, *J* = 7.2, 7.2, 7.2, 7.2, 7.2 Hz, 2 H), 1.13 (dd, *J* = 7.2, 7.2 Hz, 3 H); ^{13}C NMR (150 MHz, $CDCl_3$) δ 177.4, 162.9, 160.5, 160.5, 159.8, 132.2, 131.7, 131.1, 128.9, 126.0, 118.5, 109.7, 109.2, 97.4, 93.5, 70.9, 69.2, 22.3, 10.5.

CPD-13: mp 117.9 °C; ^{1}H NMR (600 MHz, $CDCl_3$) δ 7.91-7.84 (m, 2 H), 7.54-7.46 (m, 3 H), 6.62 (s, 1 H), 6.55 (d, *J* = 2.4 Hz, 1 H), 6.40 (d, *J* = 2.4 Hz, 1 H), 6.08 (dddd, *J* = 17.4, 10.2, 6.0, 6.0 Hz, 1 H), 5.47 (dddd, *J* = 17.4, 1.8, 1.8, 1.8 Hz, 1 H), 5.36 (dddd, *J* = 10.8, 1.2, 1.2, 1.2 Hz, 1 H), 4.63 (ddd, *J* = 5.4, 1.8, 1.8 Hz, 2 H), 4.07 (dd, *J* = 6.6, 6.6 Hz, 2 H), 1.97-1.87 (m, 2 H), 1.64-1.56 (m, 2 H), 1.00 (dd, *J* = 7.2, 7.2 Hz, 3 H); ^{13}C NMR (150 MHz, $CDCl_3$) δ 177.4, 162.9, 160.6, 160.5, 159.8, 132.2, 131.7, 131.1, 128.9, 126.0, 118.5, 109.7, 109.2, 97.4, 93.5, 69.2, 30.9, 19.2, 13.8.

References

1. For **CPD 2**: Daskiewicz, J.B.; Depeint, F.; Viornery, L.; Bayet, C.; Comte-Sarrazin, G.; Comte, G.; Gee, J.M.; Johnson, I.T.; Ndjoko, K.; Hostettmann, K.; Barron, D. Effects of Flavonoids on Cell Proliferation and Caspase Activation in a Human Colonic Cell Line HT29: An SAR Study. *J. Med. Chem.* **2005**, *48*, 2790–2804.

2. For **CPD 3**: Moreira, J.; Ribeiro, D.; Silva, P.M. A.; Nazareth, N.; Monteiro, M.; Palmeira, A.; Saraiva, L.; Pinto, M.; Bousbaa, H.; Cidade, H. New Alkoxy Flavone Derivatives Targeting Caspases: Synthesis and Antitumor Activity Evaluation. *Molecules* **2018**, *24*, 129.

3. For **CPD 4**: Khanapur, M.; Pinna, N.K.; Badiger, J. Synthesis and anti-inflammatory in vitro, in silico, and in vivo studies of flavone analogues. *Med. Chem. Res.* **2015**, *24*, 2656–2669.

4. For **CPD 5**: Kim, H.; Lim, D.; Shin, I.; Lee, D. Gram-scale synthesis of anti-pancreatic flavonoids (±)-8-[1-(4'-hydroxy-3'-methoxyphenyl)prop-2-en-1-yl]-chrysin and -galangin.*Tetrahedron* **2014**, *70*, 4738–4744.

5. For **CPD 6**: Aneja, R.; Khanna, R. N.; Seshadri, T. R. 6-Methoxyfuroflavone, a new component of the seeds of *Pongamia glabra. J. Chem. Soc.* **1963**, 163–168.

6. For **CPD 7**: Khanna, R. N.; Seshadri, T. R. Synthesis of isomers of kanjone. *Indian. J. Chem.*, **1963**, *1*, 385–387.

7. For **CPD 8**: a) Hwang, S. H.; Kim, H. Y.; Zuo, G.; Wang, Z.; Lee, J.-Y.; Lim, S. S. Anti-glycation, Carbonyl Trapping and Anti-inflammatory Activities of Chrysin Derivatives. *Molecules* **2018**, *23*, 1752. b) Wang, S.H.; Chen, C.H.; Lo, C.Y.; Feng, J.Z.; Lin, H.J.; Chang, P.Y.; Yang, L. L.; Chen, L.G.; Liu, Y. W.; Kuo, C. D.; Wu, J.Y. Synthesis and biological evaluation of novel 7-O-lipophilic substituted baicalein derivatives as potential anticancer agents. *Med. Chem. Comm.* **2015**, *6*, 1864–1873.

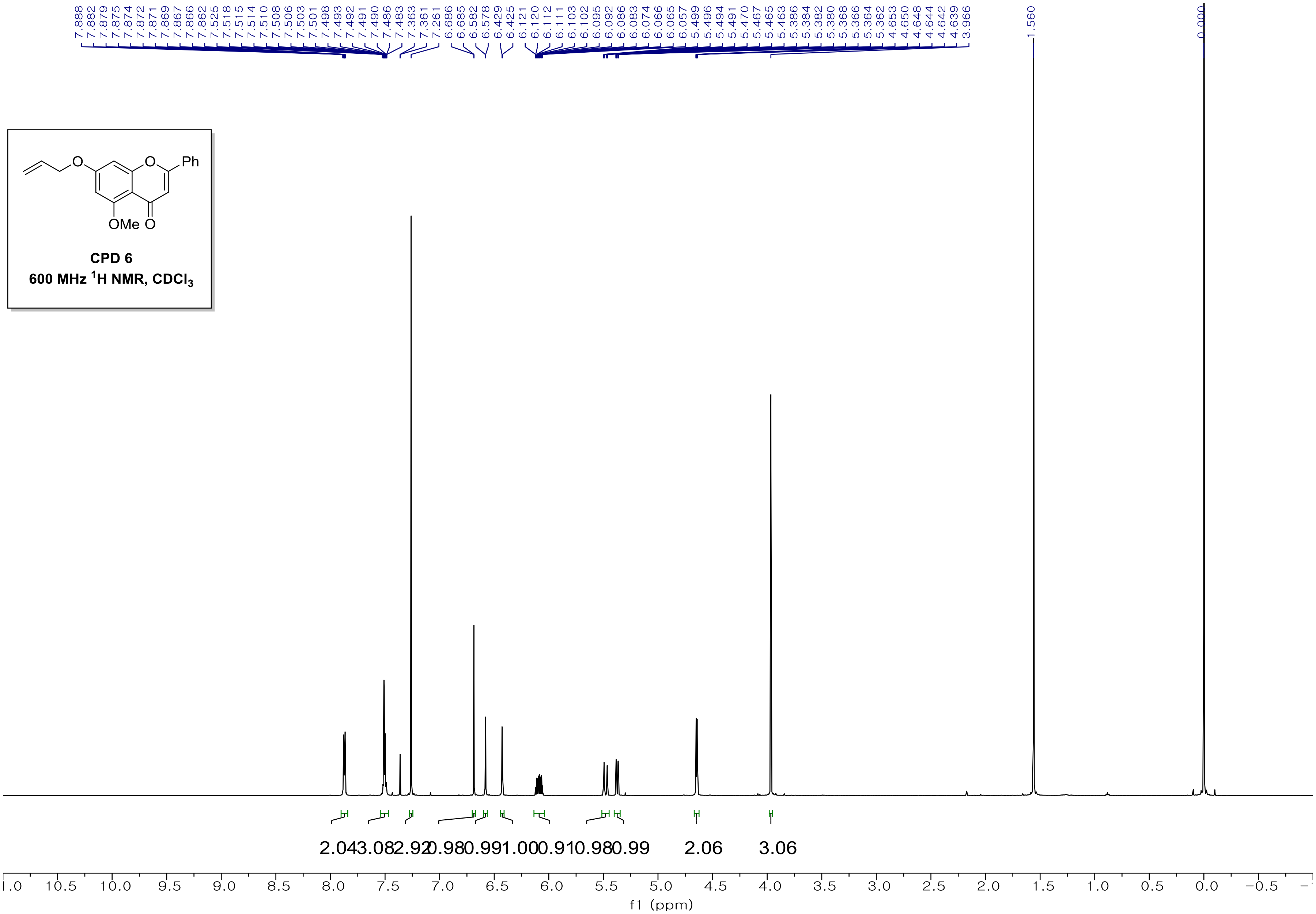
7.888 7.882 7.879 7.875 7.874 7.872 7.871 7.869 7.867 7.866 7.862 7.525 7.518 7.515 7.514 7.510 7.508 7.506 7.503 7.501 7.498 7.493 7.492 7.491 7.490 7.486 7.483 7.363 7.361 7.261 6.686 6.685 6.582 6.578 6.429 6.425 6.121 6.120 6.112 6.111 6.103 6.102 6.095 6.092 6.086 6.083 6.074 6.066 6.065 6.057 5.499 5.496 5.494 5.491 5.470 5.467 5.465 5.463 5.386 5.384 5.382 5.380 5.368 5.366 5.364 5.362 4.653 4.650 4.648 4.644 4.642 4.639 3.966
1.560
0.000
O
O
Ph
OMe
O
CPD 6
600 MHz ¹H NMR, CDCl₃
2.04 3.08 2.92 0.98 0.99 1.00 0.91 0.98 0.99
2.06
3.06
10.5 10.0 9.5 9.0 8.5 8.0 7.5 7.0 6.5 6.0 5.5 5.0 4.5 4.0 3.5 3.0 2.5 2.0 1.5 1.0 0.5 0.0 −0.5
f1 (ppm)

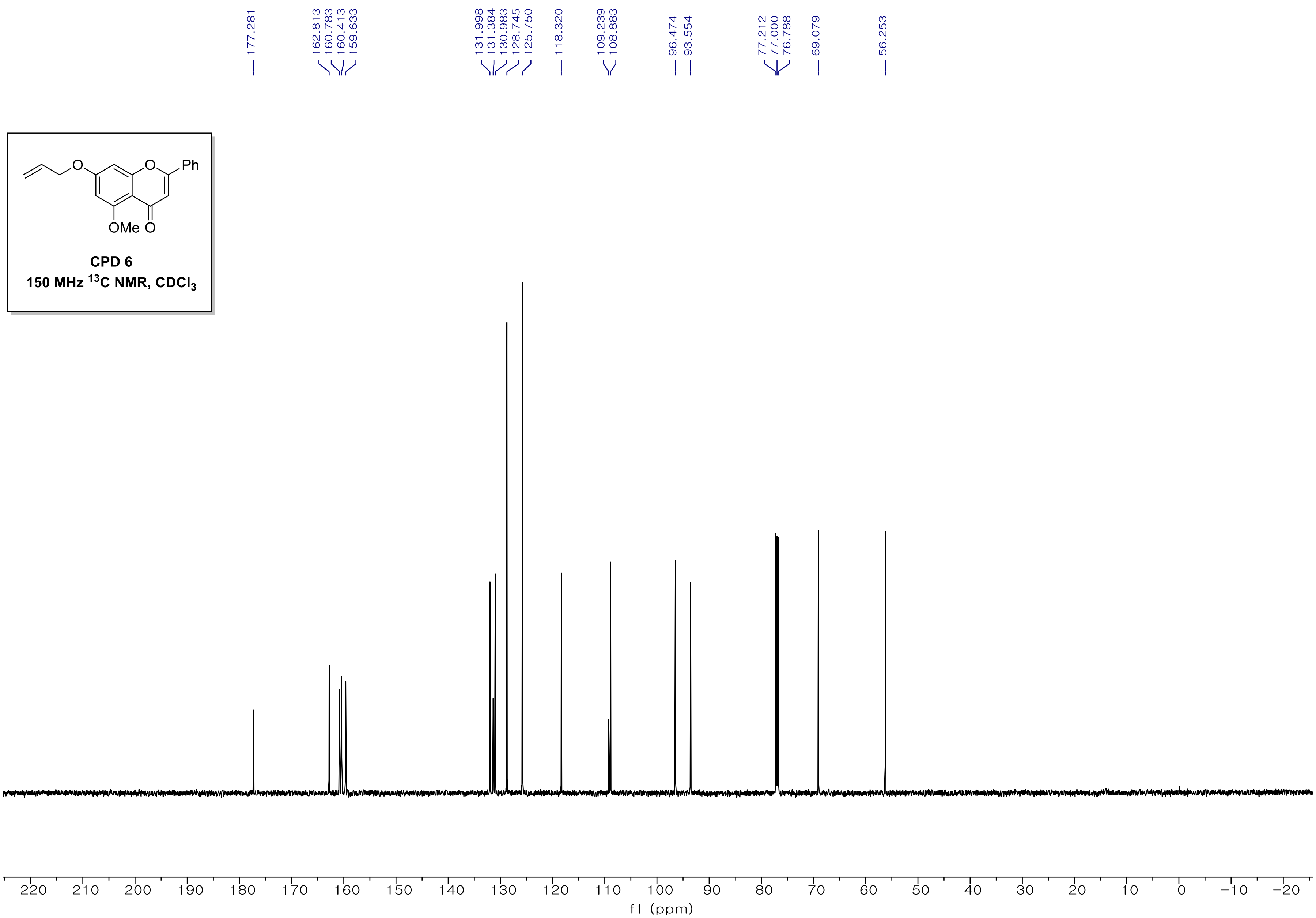

177.281
162.813
160.783
160.413
159.633
131.998
131.384
130.983
128.745
125.750
118.320
109.239
108.883
96.474
93.554
77.212
77.000
76.788
69.079
56.253
Ph
OMe
CPD 6
150 MHz ^{13}C NMR, $CDCl_3$
f1 (ppm)

State Parameter Editor

Ion Source:	Turbo Spray
Ion Source Temperature Reached	
Curtain Gas (CUR):	10.0
IonSpray Voltage (IS):	5500.0
Temperature (TEM):	0.0
Ion Source Gas 1 (GS1):	12.0
Ion Source Gas 2 (GS2):	0.0
Interface Heater (ihe):	On
Declustering Potential (DP):	70.0
Entrance Potential (EP):	10.0
Q1 Resolution:	Unit
Ion Energy 1 (IE1):	1.0
Deflector (DF):	-200.0
CEM (CEM):	2500.0

Mass Spectrometer Method Properties

```
Period 1:
--------------
Scans in Period:        594
Relative Start Time:    0.00 msec
Experiments in Period:  1

Period 1  Experiment   1:
----------------------------
Scan Type:              Q1 MS (Q1)
Polarity:               Positive
Scan Mode:              Profile
Ion Source:             Turbo Spray
Resolution Q1:          Unit
Intensity Thres.:       0.00 cps
Settling Time:          0.0000 msec
MR Pause:               5.0070 msec
MCA:                    Yes
Center/Width:           No
Step Size:              0.10 Da

Start (Da)  Stop (Da)  Time (sec)  Param  Start  Stop
100.00      500.00     0.50        CEP    7.84   19.33
Parameter Table(Period 1  Experiment   1):
CUR:        10.00
IS:         5500.00
TEM:        0.00
GS1:        12.00
GS2:        0.00
ihe:        ON
DP          70.00
EP          10.00
```

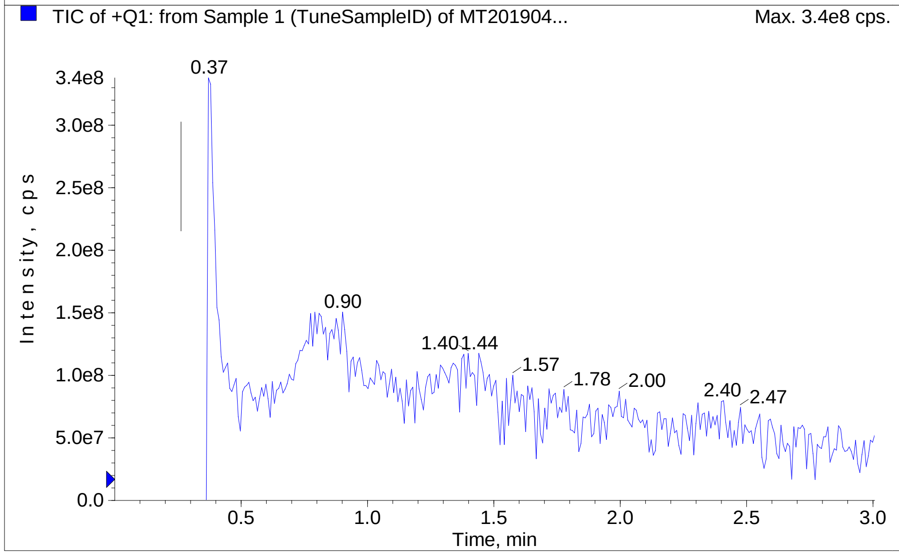

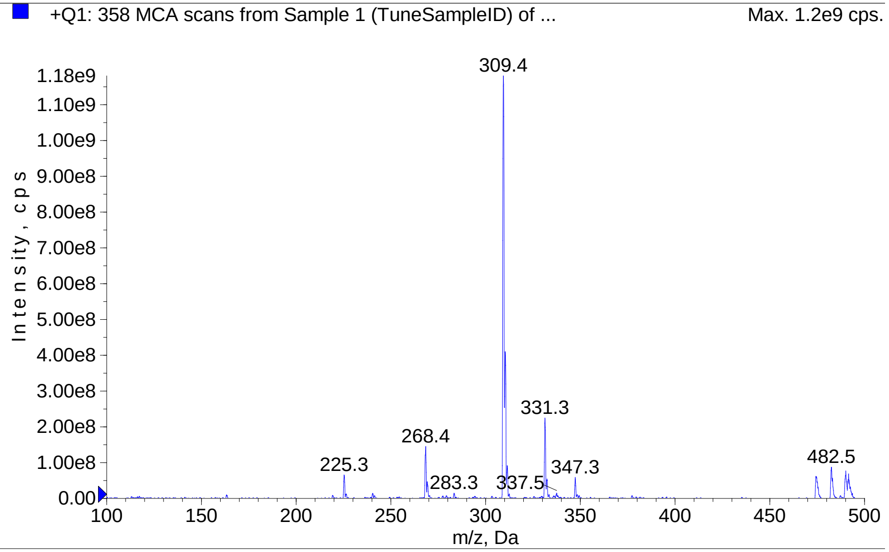

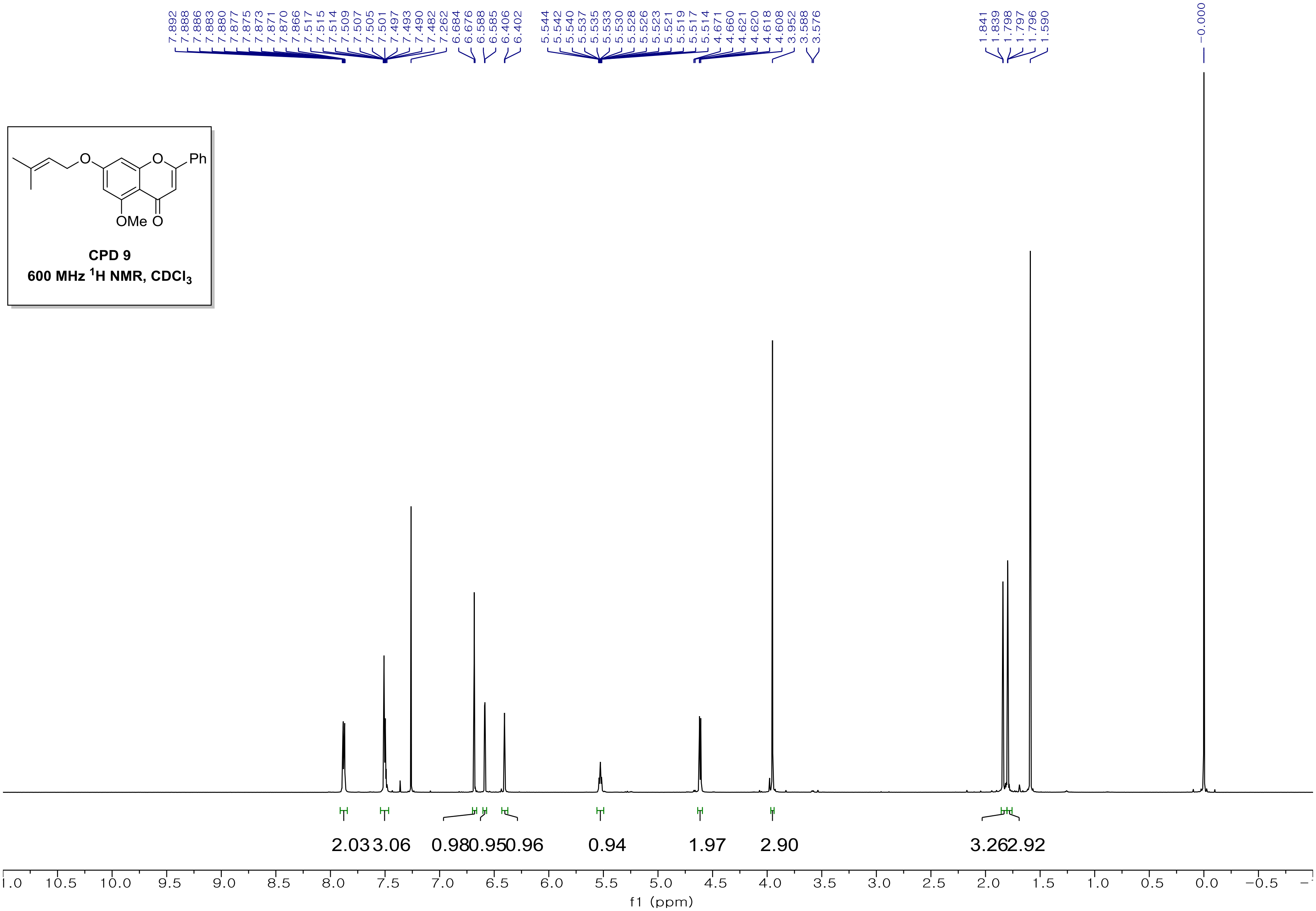
7.892 7.888 7.886 7.883 7.880 7.877 7.875 7.873 7.871 7.870 7.866 7.517 7.515 7.514 7.509 7.507 7.505 7.501 7.497 7.493 7.490 7.482 7.262 6.684 6.676 6.588 6.585 6.406 6.402
5.544 5.542 5.540 5.537 5.535 5.533 5.530 5.528 5.526 5.523 5.521 5.519 5.517 5.514 4.671 4.660 4.621 4.620 4.618 4.608 3.952 3.588 3.576
1.841 1.839 1.798 1.797 1.796 1.590
-0.000
O
O
Ph
OMe
O
CPD 9
600 MHz 1H NMR, CDCl3
2.03 3.06 0.98 0.95 0.96 0.94 1.97 2.90 3.26 2.92
f1 (ppm)

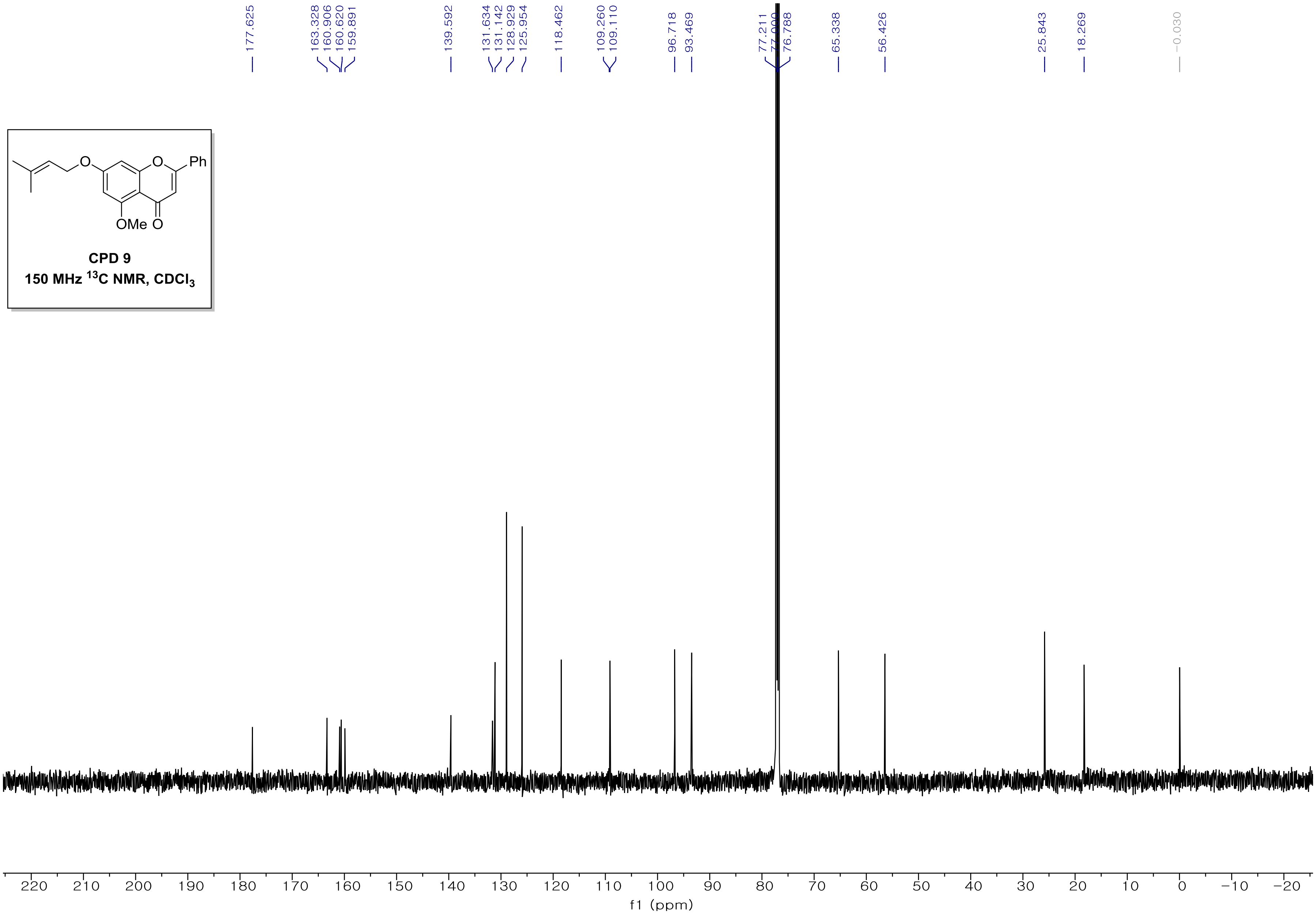
177.625
163.328
160.906
160.620
159.891
139.592
131.634
131.142
128.929
125.954
118.462
109.260
109.110
96.718
93.469
77.211
77.000
76.788
65.338
56.426
25.843
18.269
−0.030
OMe
O
Ph
CPD 9
150 MHz 13C NMR, CDCl3
220 210 200 190 180 170 160 150 140 130 120 110 100 90 80 70 60 50 40 30 20 10 0 −10 −20
f1 (ppm)

State Parameter Editor

Ion Source:	Turbo Spray
Ion Source Temperature Reached	
Curtain Gas (CUR):	10.0
IonSpray Voltage (IS):	5500.0
Temperature (TEM):	0.0
Ion Source Gas 1 (GS1):	12.0
Ion Source Gas 2 (GS2):	0.0
Interface Heater (ihe):	On
Declustering Potential (DP):	70.0
Entrance Potential (EP):	10.0
Q1 Resolution:	Unit
Ion Energy 1 (IE1):	1.0
Deflector (DF):	-200.0
CEM (CEM):	2500.0

Mass Spectrometer Method Properties

```
Period 1:
--------------
Scans in Period:        356
Relative Start Time:    0.00 msec
Experiments in Period:  1

Period 1  Experiment   1:
----------------------------
Scan Type:              Q1 MS (Q1)
Polarity:               Positive
Scan Mode:              Profile
Ion Source:             Turbo Spray
Resolution Q1:          Unit
Intensity Thres.:       0.00 cps
Settling Time:          0.0000 msec
MR Pause:               5.0070 msec
MCA:                    Yes
Center/Width:           No
Step Size:              0.10 Da

Start (Da)  Stop (Da)  Time (sec)  Param | Start  Stop
100.00      500.00     0.50        CEP   | 7.84   19.33
Parameter Table(Period 1  Experiment   1):
CUR:        10.00
TEM:        0.00
GS1:        12.00
GS2:        0.00
ihe:        ON
IS:         5500.00
DP          70.00
EP          10.00
```

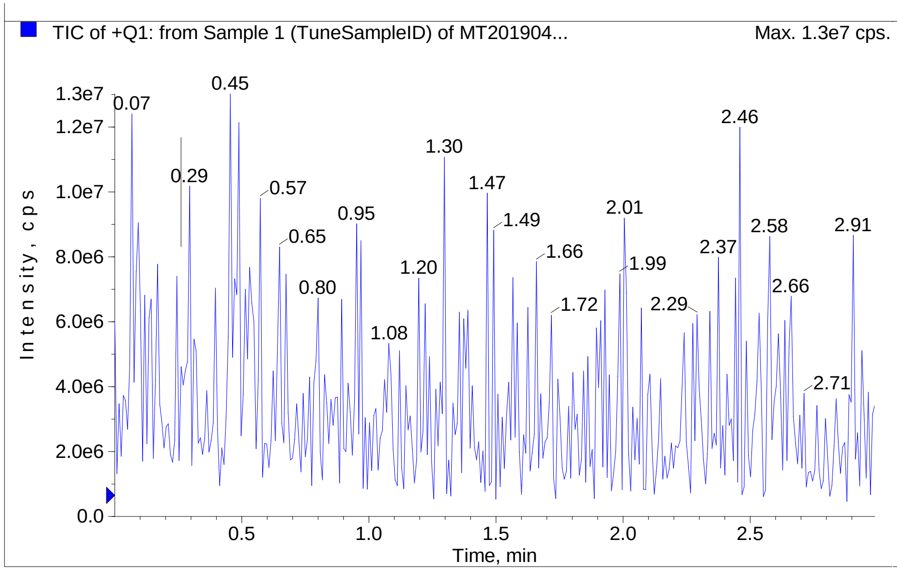

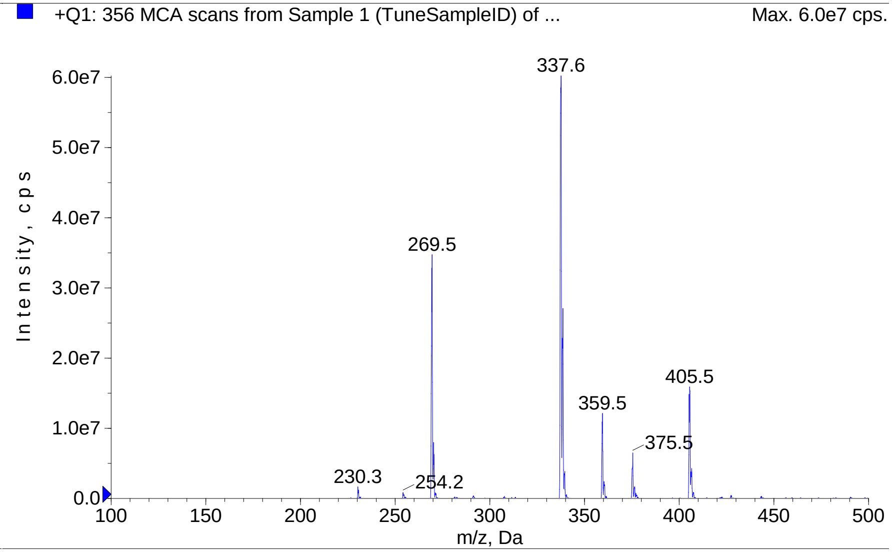

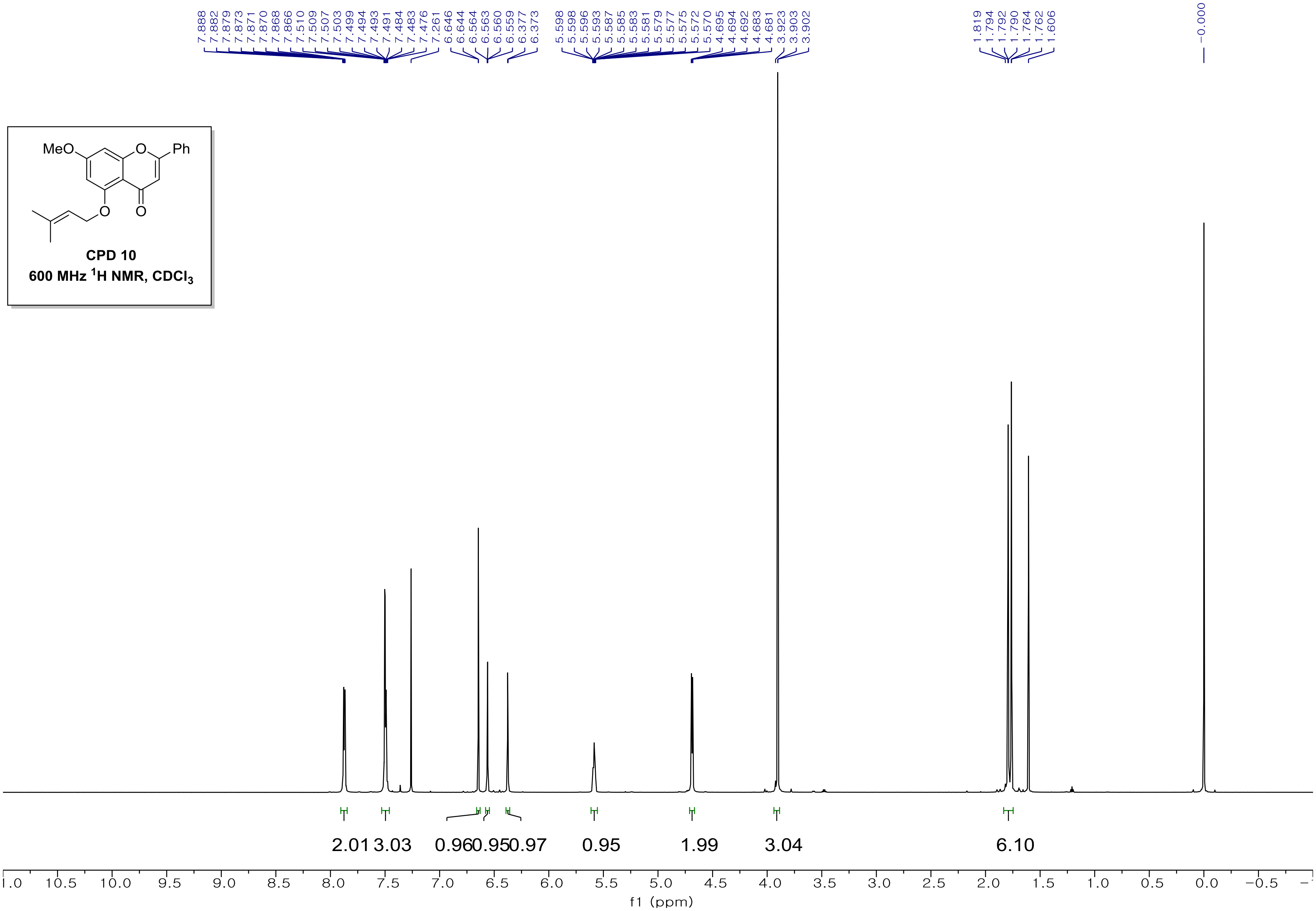
7.888
7.882
7.879
7.873
7.871
7.870
7.868
7.866
7.510
7.509
7.507
7.503
7.499
7.494
7.493
7.491
7.484
7.483
7.476
7.261
6.646
6.644
6.564
6.563
6.560
6.559
6.377
6.373
5.598
5.598
5.596
5.593
5.587
5.585
5.583
5.581
5.579
5.577
5.575
5.572
5.570
4.695
4.694
4.692
4.683
4.681
3.923
3.903
3.902
1.819
1.794
1.792
1.790
1.764
1.762
1.606
−0.000
MeO
O
Ph
O
O
CPD 10
600 MHz 1H NMR, CDCl3
2.01
3.03
0.96
0.95
0.97
0.95
1.99
3.04
6.10
10.5
10.0
9.5
9.0
8.5
8.0
7.5
7.0
6.5
6.0
5.5
5.0
4.5
4.0
3.5
3.0
2.5
2.0
1.5
1.0
0.5
0.0
−0.5
f1 (ppm)

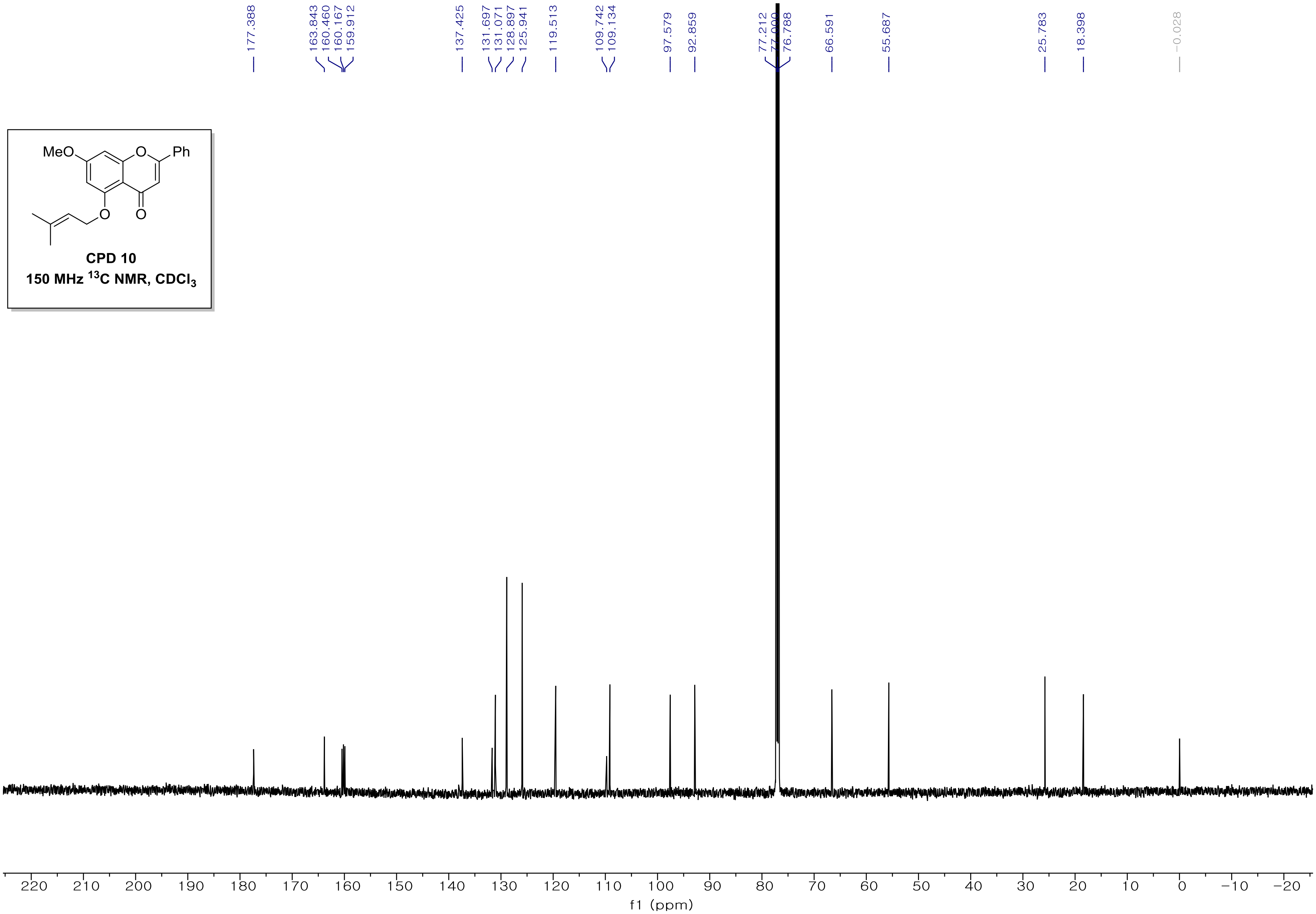
177.388
163.843
160.460
160.167
159.912
137.425
131.697
131.071
128.897
125.941
119.513
109.742
109.134
97.579
92.859
77.212
76.788
66.591
55.687
25.783
18.398
−0.028
MeO
O
Ph
O
O
CPD 10
150 MHz 13C NMR, CDCl3
220 210 200 190 180 170 160 150 140 130 120 110 100 90 80 70 60 50 40 30 20 10 0 −10 −20
f1 (ppm)

State Parameter Editor

Ion Source:	Turbo Spray
Ion Source Temperature Reached	
Curtain Gas (CUR):	10.0
IonSpray Voltage (IS):	5500.0
Temperature (TEM):	0.0
Ion Source Gas 1 (GS1):	12.0
Ion Source Gas 2 (GS2):	0.0
Interface Heater (ihe):	On
Declustering Potential (DP):	70.0
Entrance Potential (EP):	10.0
Q1 Resolution:	Unit
Ion Energy 1 (IE1):	1.0
Deflector (DF):	-200.0
CEM (CEM):	2500.0

Mass Spectrometer Method Properties

```
Period 1:
--------------
Scans in Period:        356
Relative Start Time:    0.00 msec
Experiments in Period:  1

Period 1  Experiment   1:
----------------------------
Scan Type:              Q1 MS (Q1)
Polarity:               Positive
Scan Mode:              Profile
Ion Source:             Turbo Spray
Resolution Q1:          Unit
Intensity Thres.:       0.00 cps
Settling Time:          0.0000 msec
MR Pause:               5.0070 msec
MCA:                    Yes
Center/Width:           No
Step Size:              0.10 Da

Start (Da)  Stop (Da)  Time (sec)  Param  Start  Stop
100.00      500.00     0.50        CEP    7.84   19.33
Parameter Table(Period 1  Experiment   1):
CUR:        10.00
TEM:        0.00
GS1:        12.00
GS2:        0.00
ihe:        ON
IS:         5500.00
DP          70.00
EP          10.00
```

TIC of +Q1: from Sample 1 (TuneSampleID) of MT201904... Max. 6.4e7 cps.

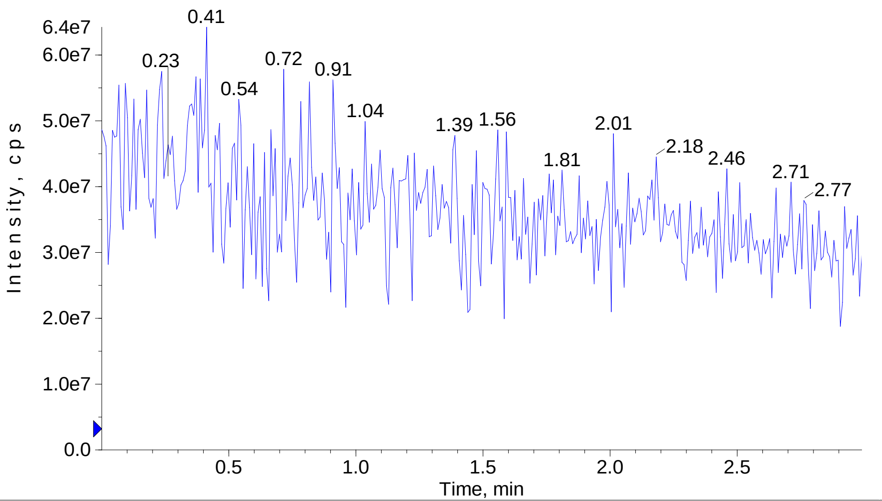

+Q1: 356 MCA scans from Sample 1 (TuneSampleID) of ... Max. 5.7e8 cps.

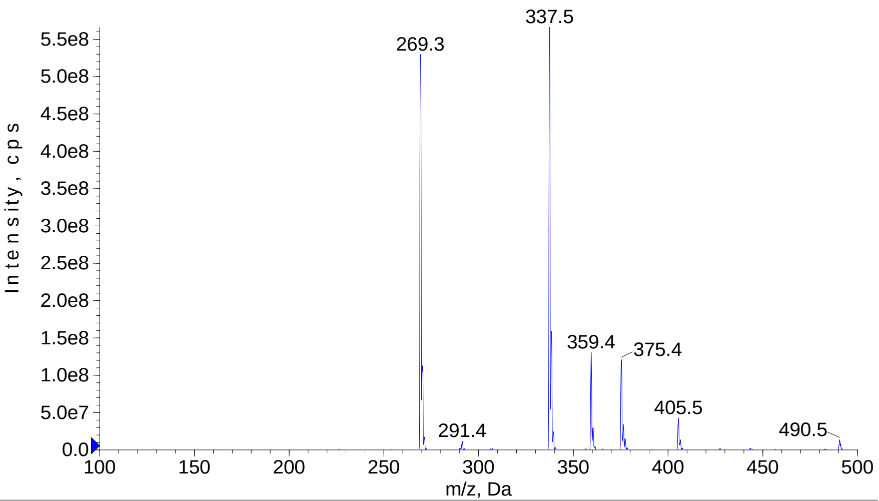

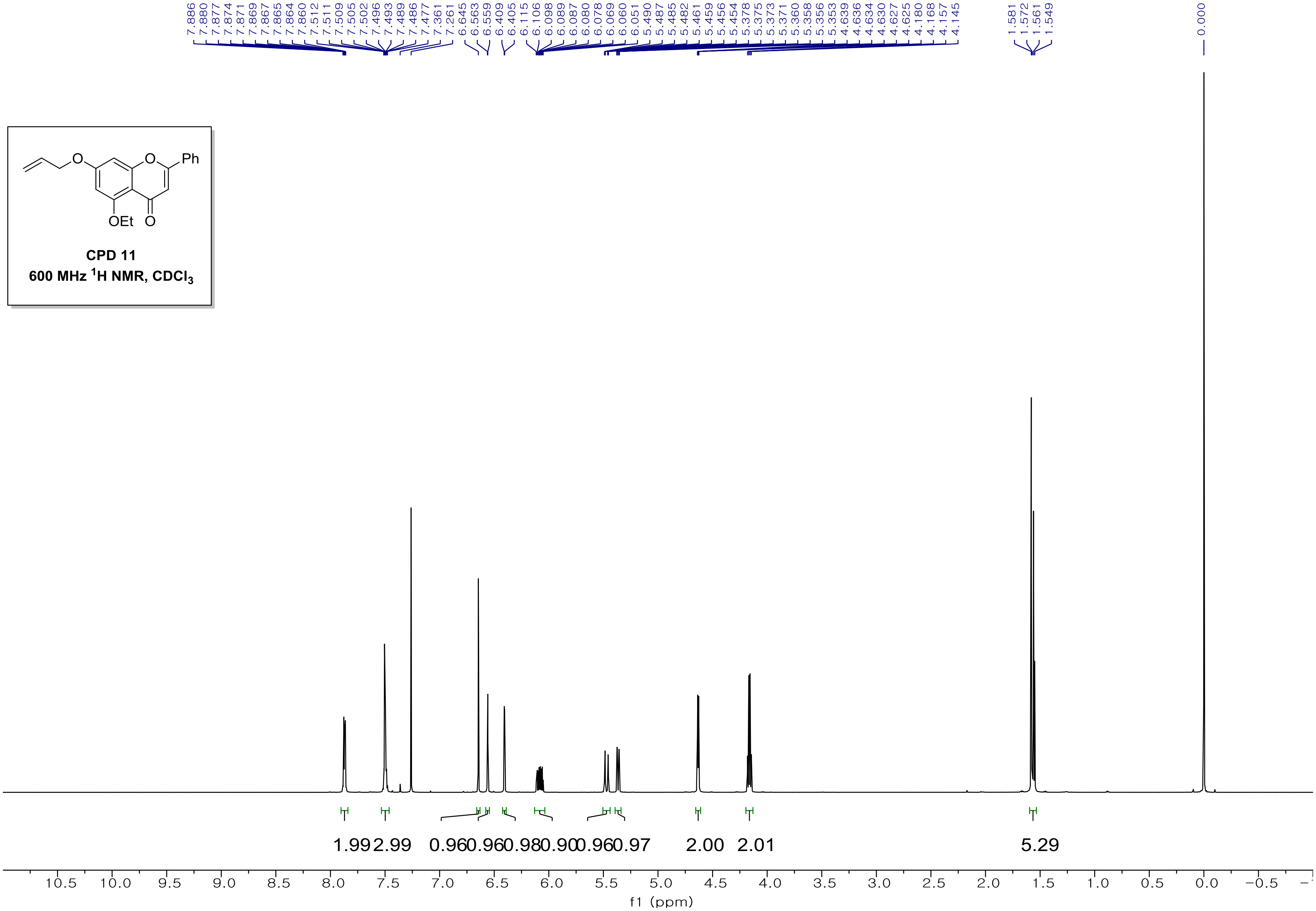
CPD 11
600 MHz 1H NMR, CDCl3
f1 (ppm)

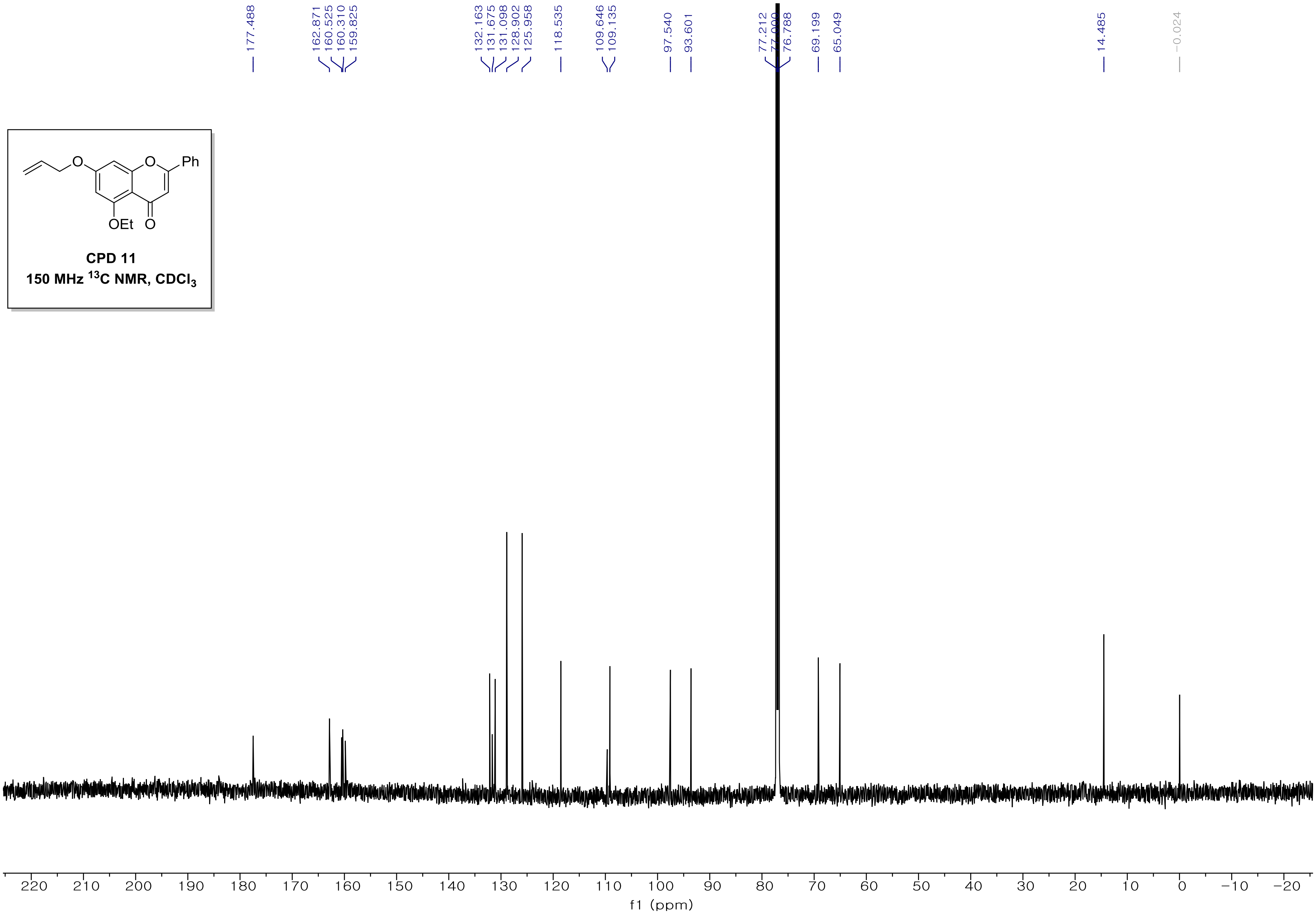
177.488
162.871
160.525
160.310
159.825
132.163
131.675
131.098
128.902
125.958
118.535
109.646
109.135
97.540
93.601
77.212
77.000
76.788
69.199
65.049
14.485
−0.024
Ph
OEt
CPD 11
150 MHz 13C NMR, CDCl3
220 210 200 190 180 170 160 150 140 130 120 110 100 90 80 70 60 50 40 30 20 10 0 −10 −20
f1 (ppm)

State Parameter Editor

Ion Source:	Turbo Spray
Ion Source Temperature Reached	
Curtain Gas (CUR):	10.0
IonSpray Voltage (IS):	5500.0
Temperature (TEM):	0.0
Ion Source Gas 1 (GS1):	12.0
Ion Source Gas 2 (GS2):	0.0
Interface Heater (ihe):	On
Declustering Potential (DP):	70.0
Entrance Potential (EP):	10.0
Q1 Resolution:	Unit
Ion Energy 1 (IE1):	1.0
Deflector (DF):	-200.0
CEM (CEM):	2500.0

Mass Spectrometer Method Properties

```
Period 1:
--------------
Scans in Period:        356
Relative Start Time:    0.00 msec
Experiments in Period:  1

Period 1  Experiment   1:
----------------------------
Scan Type:              Q1 MS (Q1)
Polarity:               Positive
Scan Mode:              Profile
Ion Source:             Turbo Spray
Resolution Q1:          Unit
Intensity Thres.:       0.00 cps
Settling Time:          0.0000 msec
MR Pause:               5.0070 msec
MCA:                    Yes
Center/Width:           No
Step Size:              0.10 Da

Start (Da)  Stop (Da)  Time (sec)  Param  Start  Stop
100.00      500.00     0.50        CEP    7.84   19.33
Parameter Table(Period 1  Experiment   1):
CUR:        10.00
TEM:        0.00
GS1:        12.00
GS2:        0.00
ihe:        ON
IS:         5500.00
DP          70.00
EP          10.00
```

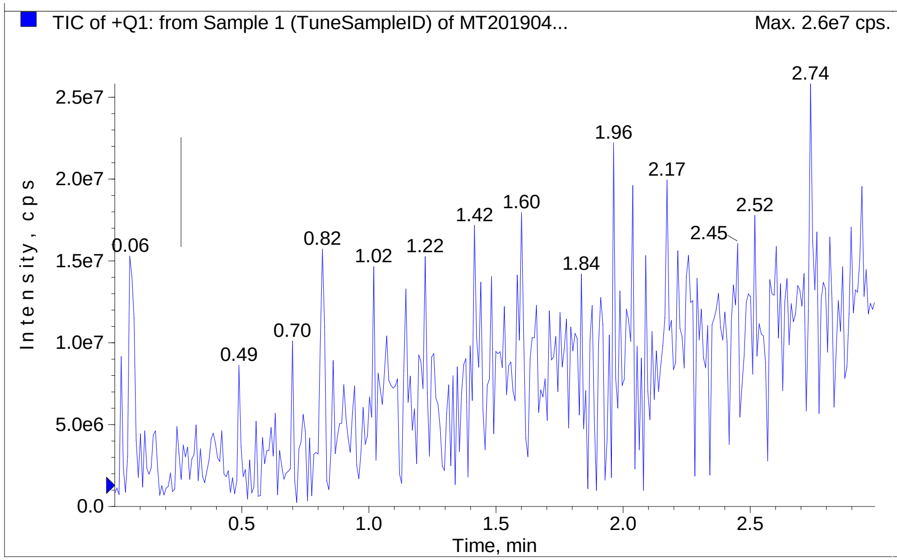

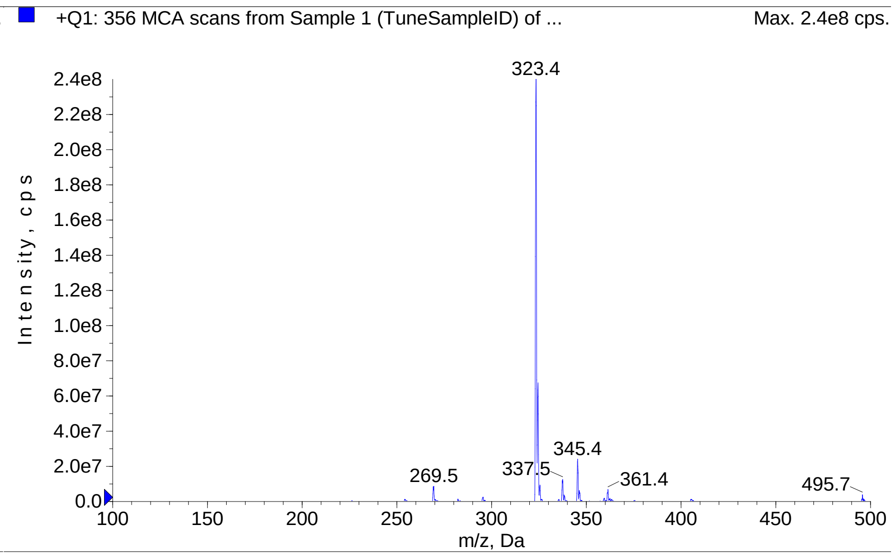

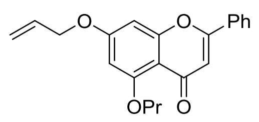

CPD 12

600 MHz ¹H NMR, $CDCl_3$

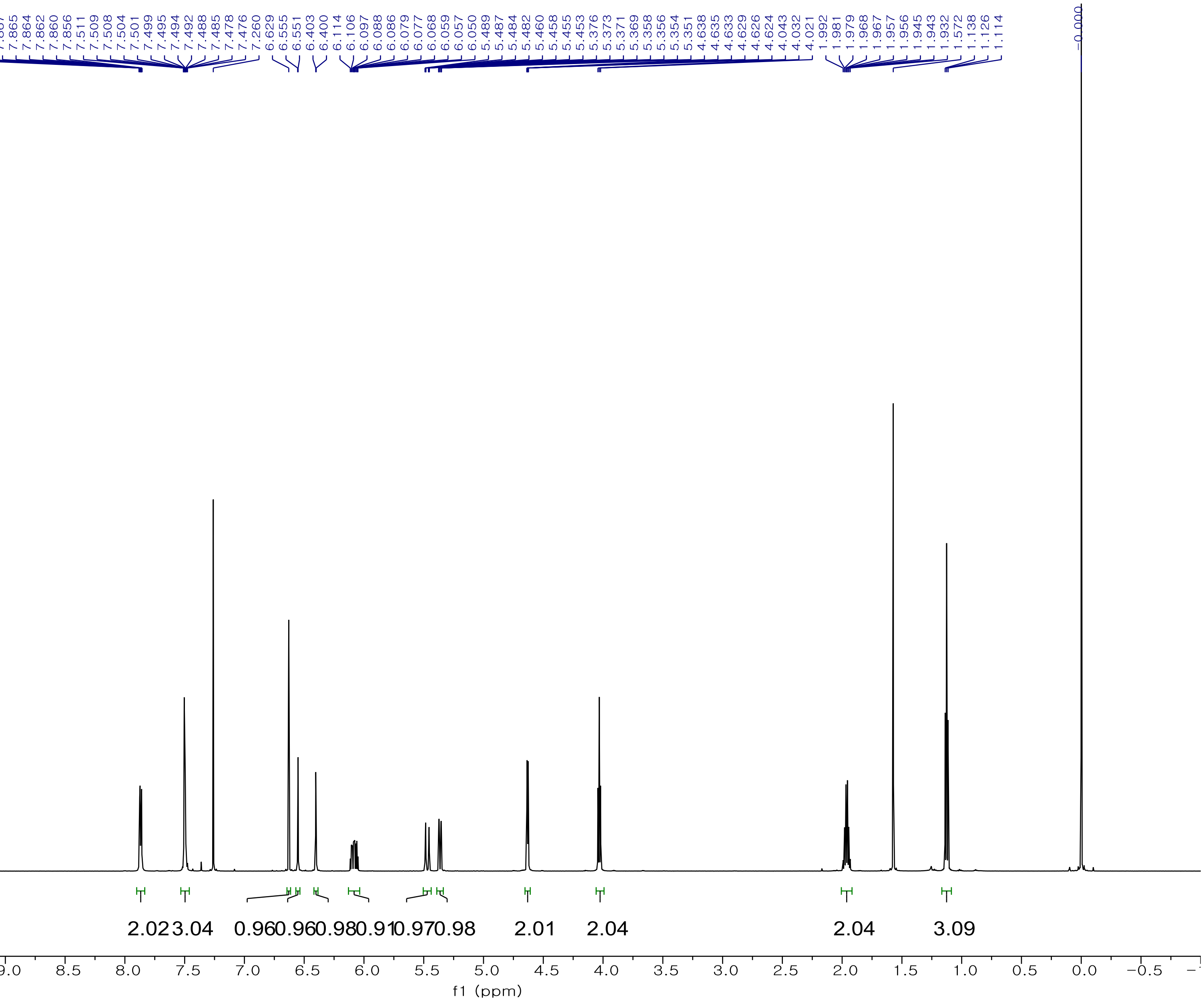

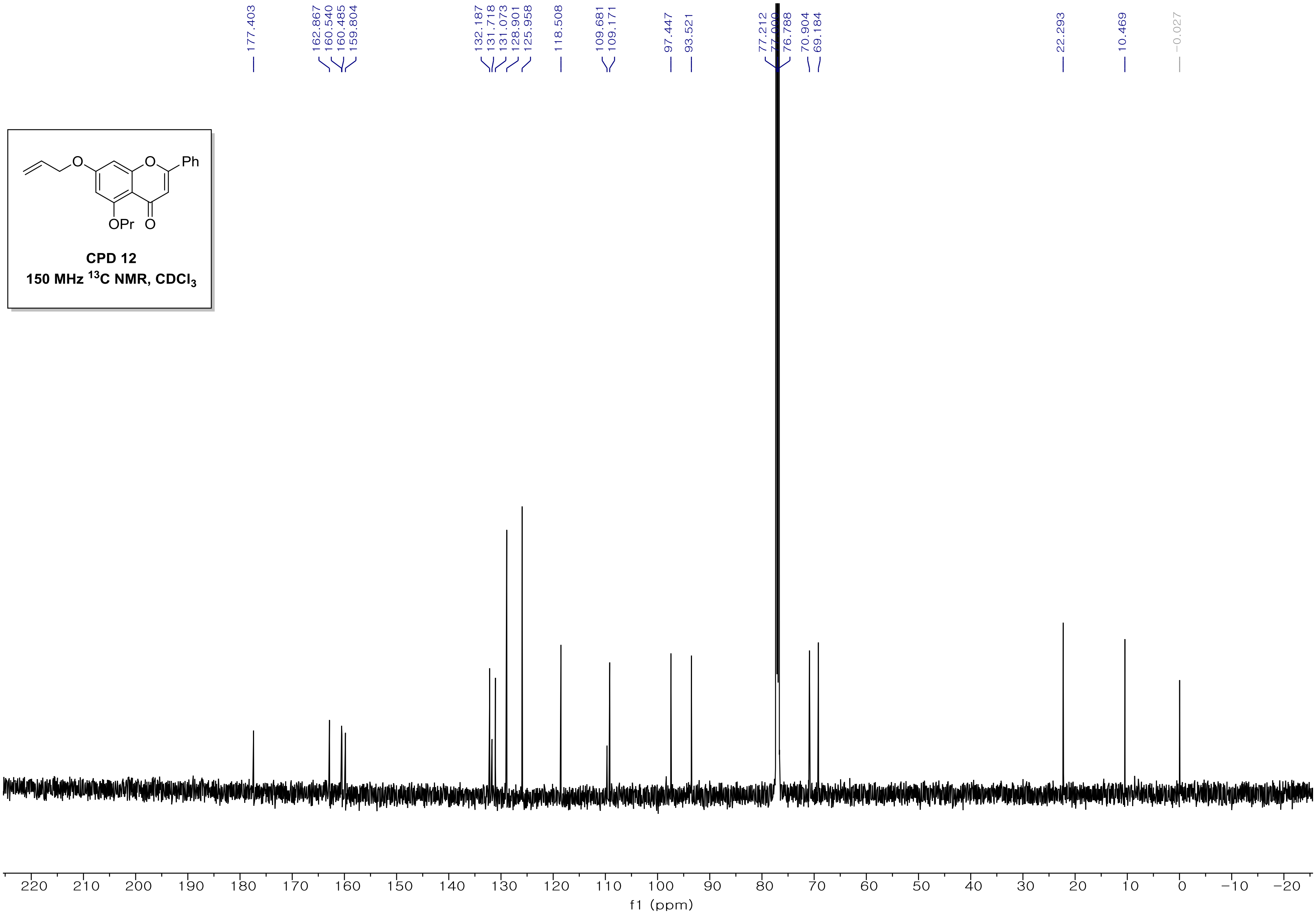
177.403
162.867
160.540
160.485
159.804
132.187
131.718
131.073
128.901
125.958
118.508
109.681
109.171
97.447
93.521
77.212
76.788
70.904
69.184
22.293
10.469
−0.027
O
O
Ph
OPr
O
CPD 12
150 MHz 13C NMR, CDCl3
220 210 200 190 180 170 160 150 140 130 120 110 100 90 80 70 60 50 40 30 20 10 0 −10 −20
f1 (ppm)

State Parameter Editor

Ion Source:	Turbo Spray
Ion Source Temperature Reached	
Curtain Gas (CUR):	10.0
IonSpray Voltage (IS):	5500.0
Temperature (TEM):	0.0
Ion Source Gas 1 (GS1):	12.0
Ion Source Gas 2 (GS2):	0.0
Interface Heater (ihe):	On
Declustering Potential (DP):	70.0
Entrance Potential (EP):	10.0
Q1 Resolution:	Unit
Ion Energy 1 (IE1):	1.0
Deflector (DF):	-200.0
CEM (CEM):	2500.0

Mass Spectrometer Method Properties

```
Period 1:
--------------
Scans in Period:         356
Relative Start Time:     0.00 msec
Experiments in Period:   1

Period 1  Experiment   1:
----------------------------
Scan Type:               Q1 MS (Q1)
Polarity:                Positive
Scan Mode:               Profile
Ion Source:              Turbo Spray
Resolution Q1:           Unit
Intensity Thres.:        0.00 cps
Settling Time:           0.0000 msec
MR Pause:                5.0070 msec
MCA:                     Yes
Center/Width:            No
Step Size:               0.10 Da

Start (Da)  Stop (Da)  Time (sec)  Param  Start  Stop
100.00      500.00     0.50        CEP    7.84   19.33
Parameter Table(Period 1  Experiment   1):
CUR:        10.00
TEM:        0.00
GS1:        12.00
GS2:        0.00
ihe:        ON
IS:         5500.00
DP          70.00
EP          10.00
```

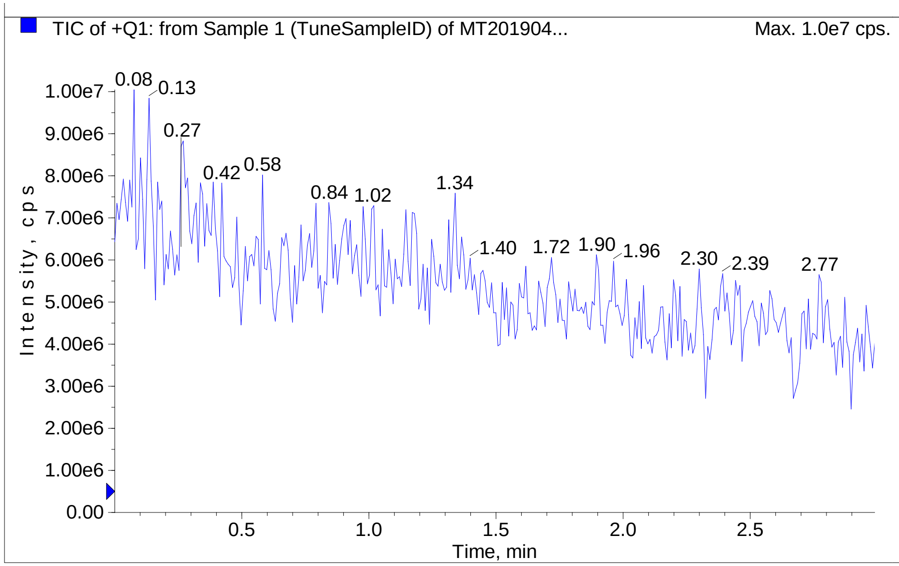

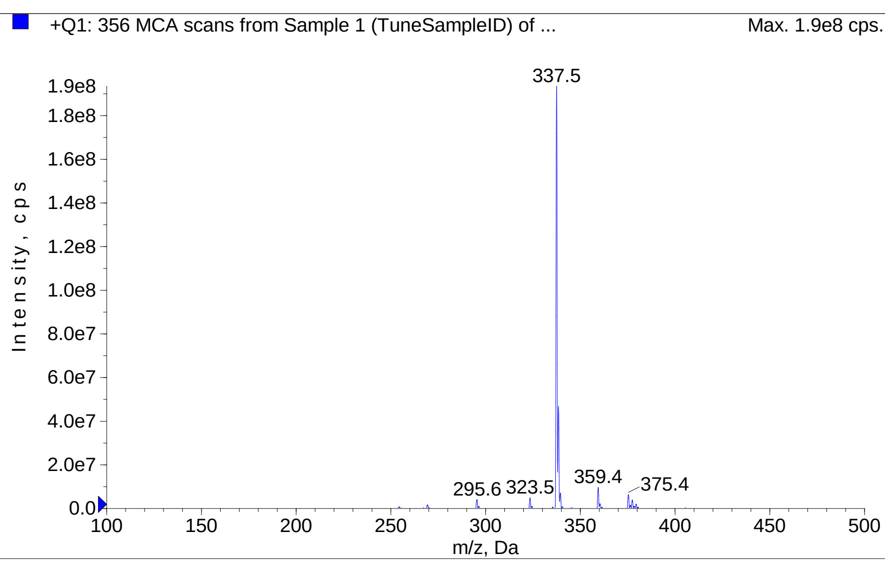

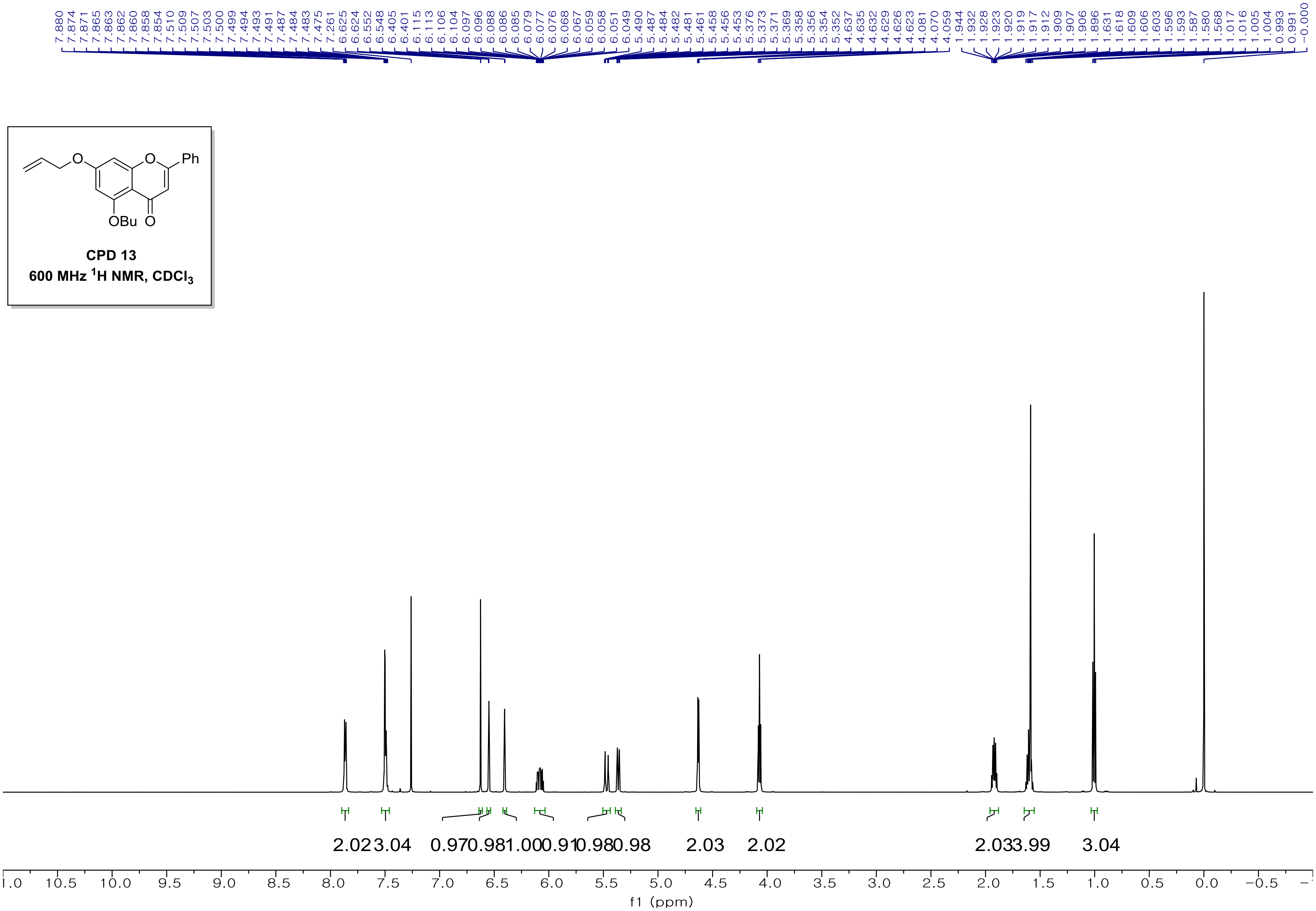

CPD 13
600 MHz 1H NMR, CDCl3

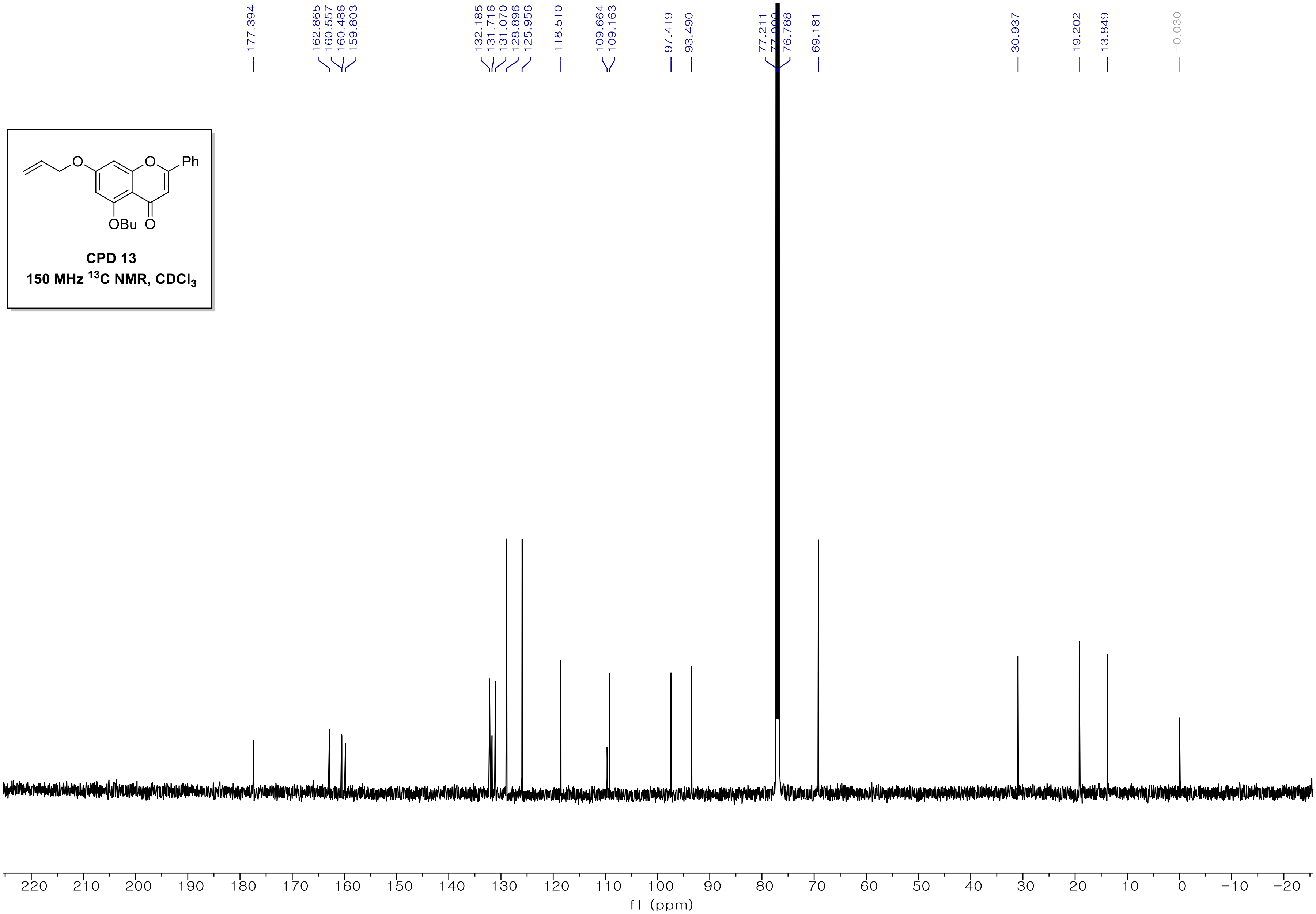

177.394
162.865
160.557
160.486
159.803
132.185
131.716
131.070
128.896
125.956
118.510
109.664
109.163
97.419
93.490
77.211
77.000
76.788
69.181
30.937
19.202
13.849
−0.030
OBu
Ph
CPD 13
150 MHz 13C NMR, CDCl3
f1 (ppm)

State Parameter Editor

Ion Source:	Turbo Spray
Ion Source Temperature Reached	
Curtain Gas (CUR):	10.0
IonSpray Voltage (IS):	5500.0
Temperature (TEM):	0.0
Ion Source Gas 1 (GS1):	12.0
Ion Source Gas 2 (GS2):	0.0
Interface Heater (ihe):	On
Declustering Potential (DP):	70.0
Entrance Potential (EP):	10.0
Q1 Resolution:	Unit
Ion Energy 1 (IE1):	1.0
Deflector (DF):	-200.0
CEM (CEM):	2500.0

Mass Spectrometer Method Properties

```
Period 1:
--------------
Scans in Period:        356
Relative Start Time:    0.00 msec
Experiments in Period:  1

Period 1  Experiment   1:
----------------------------
Scan Type:              Q1 MS (Q1)
Polarity:               Positive
Scan Mode:              Profile
Ion Source:             Turbo Spray
Resolution Q1:          Unit
Intensity Thres.:       0.00 cps
Settling Time:          0.0000 msec
MR Pause:               5.0070 msec
MCA:                    Yes
Center/Width:           No
Step Size:              0.10 Da

Start (Da)  Stop (Da)  Time (sec)  Param  Start  Stop
100.00      500.00     0.50        CEP    7.84   19.33
Parameter Table(Period 1  Experiment   1):
CUR:        10.00
TEM:        0.00
GS1:        12.00
GS2:        0.00
ihe:        ON
IS:         5500.00
DP          70.00
EP          10.00
```

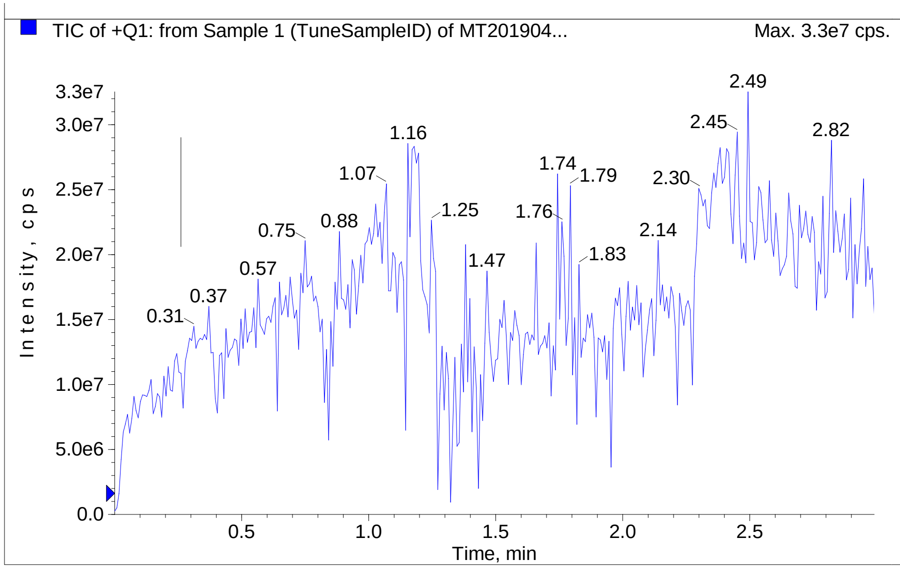

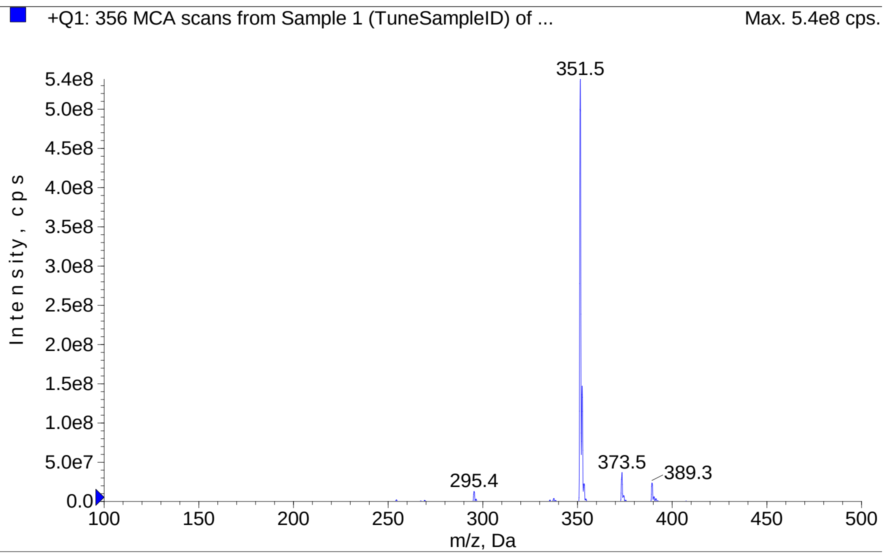

Supplement: Supplementary file 1 [file ijms-20-02607-s001.pdf]
